# Predicting disease recurrence in patients with endometriosis: an observational study

**DOI:** 10.1186/s12916-024-03508-7

**Published:** 2024-08-07

**Authors:** Sarah J. Holdsworth-Carson, Jessica Chung, Dorothy A. Machalek, Rebecca Li, Byung Kyu Jun, Meaghan J. Griffiths, Molly Churchill, Tristan McCaughey, Debbie Nisbet, Uri Dior, Jacqueline F. Donoghue, Grant W. Montgomery, Charlotte Reddington, Jane E. Girling, Martin Healey, Peter A. W. Rogers

**Affiliations:** 1 Department of Obstetrics, Gynaecology and Newborn Health, University of Melbourne and Gynaecology Research Centre, Royal Women’s Hospital, Grattan St & Flemington Rd, Parkville, VIC 3052 Australia; 2 The Julia Argyrou Endometriosis Centre, Epworth HealthCare, Ground Floor, 185-187 Hoddle Street, Richmond, VIC 3121 Australia; 3 Melbourne Bioinformatics, University of Melbourne, 21 Bedford St, North Melbourne, VIC 3051 Australia; 4 The Kirby Institute, University of New South Wales, High Street, Kensington, NSW 2052 Australia; 5 Centre for Women’s Infectious Diseases, The Royal Women’s Hospital, Grattan St & Flemington Rd, Parkville, VIC 3052 Australia; 6 Ultrasound Services, Royal Women’s Hospital, Grattan St & Flemington Rd, Parkville, VIC 3052 Australia; 7 Department of Radiology, University of Melbourne, Royal Melbourne Hospital, Royal Parade, Parkville, VIC 3050 Australia; 8 Faculty of Medicine, Department of Obstetrics and Gynaecology, Hadassah Medical Center, P.O Box 12000, Jerusalem, 91120 Israel; 9 Institute for Molecular Bioscience, University of Queensland, 306 Carmody Road, St Lucia, Brisbane, QLD 4072 Australia; 10 Department of Anatomy, School of Biomedical Sciences, University of Otago, 270 Great King Street, Dunedin, 9016 New Zealand

**Keywords:** Endometriosis, Recurrent endometriosis, Endometriosis recurrence, Repeat surgery, Reoperation

## Abstract

**Background:**

Despite surgical and pharmacological interventions, endometriosis can recur. Reliable information regarding risk of recurrence following a first diagnosis is scant. The aim of this study was to examine clinical and survey data in the setting of disease recurrence to identify predictors of risk of endometriosis recurrence.

**Methods:**

This observational study reviewed data from 794 patients having surgery for pelvic pain or endometriosis. Patients were stratified into two analytic groups based on self-reported or surgically confirmed recurrent endometriosis. Statistical analyses included univariate, followed by multivariate logistic regression to identify risk factors of recurrence, with least absolute shrinkage and selection operator (Lasso) regularisation. Risk-calibrated Supersparse Linear Integer Models (RiskSLIM) and survival analyses (with Lasso) were undertaken to identify predictive features of recurrence.

**Results:**

Several significant features were repeatedly identified in association with recurrence, including adhesions, high rASRM score, deep disease, bowel lesions, adenomyosis, emergency room attendance for pelvic pain, younger age at menarche, higher gravidity, high blood pressure and older age. In the surgically confirmed group, with a score of 5, the RiskSLIM method was able to predict the risk of recurrence (compared to a single diagnosis) at 95.3% and included adenomyosis and adhesions in the model. Survival analysis further highlighted bowel lesions, adhesions and adenomyosis.

**Conclusions:**

Following an initial diagnosis of endometriosis, clinical decision-making regarding disease management should take into consideration the presence of bowel lesions, adhesions and adenomyosis, which increase the risk of endometriosis recurrence.

## Background

The symptoms and appearance of endometriosis are heterogeneous. Furthermore, the clinical manifestation and responsiveness to treatments can change over time in individuals. This means there is no ‘one shoe fits all’ approach to managing and treating endometriosis, and in the absence of a failsafe cure, endometriosis disease recurrence is common (6–67%) [1–4]. Some previously reported risk factors for endometriosis recurrence include severe or deep disease, younger age and conservative surgery with conservation of the ovaries and uterus [2]. Apprehension regarding recurrent disease is high among patients. ‘What is the most effective way of stopping endometriosis progressing and/or spreading to other organs (eg after surgery)?’ was listed in the top 10 endometriosis research priorities by consumers and health-care professionals in the UK and Ireland in 2017 [5].

Recurrence of endometriosis in an individual following complete surgical excision can be defined as lesion recurrence on reoperation (with or without histological confirmation) or on imaging. However, symptom-based suspected recurrence, not proven by imaging and/or surgery, may also be considered a subtype of recurrent disease [6, 7]. With such broad definitions encapsulating recurrent endometriosis, it is not surprising the reported range of disease recurrence is so wide. Some limitations that prevent findings from being replicated include failure to report the duration of follow-up (or short follow-up periods), examination of specific lesions types, for example, endometrioma or deep lesions only, and failure to report if excision was complete, thus bringing into question the possibility of residual, rather than recurrent disease [8]. Hence, the determinants for recurrence have not been established and the true prevalence of endometriosis recurrence remains unknown.

To improve long-term health outcomes for patients with endometriosis, it is important that we increase our understanding of why endometriosis lesions return in some individuals and learn which risk factors contribute to the probability of the disease recurrence. The aim of this study was to identify factors that are associated with increased risk for recurrent disease, specifically lesions returning, and to help predict probable risk of endometriosis recurrence.

## Methods

### Study design and participants

This observational study was performed as a secondary analysis of a prospective study (titled ‘Cellular, Molecular and Genetic Mechanisms of Endometriosis’), was performed according to the STROBE statement [9] and was conducted with the consent of patients who were having laparoscopic surgery between May 2012 and March 2019 at a tertiary university-affiliated referral centre (Royal Women’s Hospital [RWH], Melbourne, Australia). In this paper, the index surgery was considered to be the surgery for which recruitment into the study occurred. The study was approved by the Royal Women’s Hospital Human Research Ethics Committee (Project #10-43, #11-24 and #16-43), which operates in accordance with the National Health and Medical Research Council (NHMRC) National Statement on Ethical Conduct in Human Research.

The total number of patients available for inclusion in this study was *n* = 794. Patients were eligible for recruitment if they were English speaking, aged ≥ 18 years, pre-menopausal, not pregnant and were undergoing laparoscopic surgery for investigation of pelvic pain and/or treatment of endometriosis, usually with hysteroscopy, dilation and curettage (unless prior hysterectomy) and cystoscopy. Medical information (including age, blood pressure [BP] and body mass index [BMI]), surgical reports, ultrasound reports, revised American Society for Reproductive Medicine (rASRM) endometriosis scores [10] and pathology findings were collected. Surgeons also filled out a study document providing further details on lesions, including location (recorded as pouch of Douglas, uterovesical (UV) fold, bladder, bowel, fallopian tube, pelvic side wall, pararectal space, uterosacral ligament or other) and adhesions (presence yes/no). At each surgery, there was at least one surgeon with extensive experience with laparoscopic treatment of endometriosis. All surgical reports were independently checked by a senior specialist laparoscopic surgeon (M.H.). Information collected from ultrasound reports included uterine volume, uterine position, presence of fibroids (including number of fibroids and volume of the largest fibroid), ovarian cysts, polycystic ovaries and adenomyosis (including linear striations, heterogeneous myometrium and thickened posterior wall). All collected ultrasound data were overseen by a senior ultrasound specialist (D.N.).

Participants completed a pre-surgery questionnaire covering a range of self-reported gynaecological and non-gynaecological variables. Gynaecological-related variables included the following: a prior diagnosis of endometriosis (prior to this index surgery), family history of endometriosis, age at menarche and pelvic pain symptoms (dysmenorrhea, non-cyclical pelvic pain, dyspareunia or need to seek emergency room [ER] treatment because of pain [prior to this index surgery]). Patients reported pregnancies (gravidity), births (parity), current hormone contraceptive use at the time of completing the pre-surgery survey, previous diagnosis of ovarian cysts and polycystic ovary disease, uterine fibroids, fibrotic breasts, adenomyosis, pre-cancer of the cervix (or an abnormal Papanicolaou test) and prior hysterectomy. Allergic disorders commonly associated with endometriosis [11–13] were also recorded (presence of general food allergies or intolerances, taste or smell disturbances, hay fever and eczema). Patients were also asked to provide tobacco smoking status (current, past or never).

**Fig. 1:**
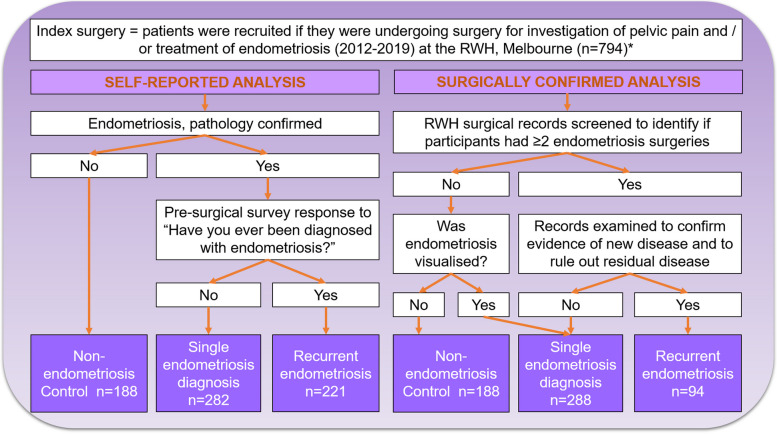
Flow chart illustrating the selection of participants in each analytic cohort. *There were *n* = 794 patients for inclusion in this study. Each analytic cohort was conducted independently; self-reported analysis or surgically confirmed analysis, and subjects crossed over into both cohorts

Patients were stratified into two analytic groups based on self-reported or surgically recorded recurrent endometriosis (Fig. 1):

- Self-reported analysis

Patients who received a positive, pathology-confirmed, endometriosis diagnosis at the referral centre (RWH) were classified as having a single endometriosis diagnosis or recurrent endometriosis based on their survey response to ‘Have you ever been diagnosed with endometriosis?’ (Yes = recurrent endometriosis; *n* = 221 or No = single diagnosis of endometriosis; *n* = 302). Those classified as a single diagnosis were further screened using hospital records, if they were found to have a diagnosis of endometriosis following a different surgery they were removed (final number; *n* = 282). Those without any record of surgically or pathology-confirmed endometriosis were classified as non-endometriosis controls (records were also screened for evidence of endometriosis from other laparoscopic surgeries) (*n* = 188).

- Surgically confirmed recurrence analysis

The hospital surgical records (RWH) of all participants were screened to identify if participants had undergone more than one surgery for endometriosis. For each potential case of recurrent endometriosis, the surgical reports were vigorously examined to identify presence and location of any residual disease at the surgery. As no standardised format for recording untreated disease exists in the surgical report format, complete text review was undertaken of the reports (by T.M.). In the event of known residual disease (disease recorded as left behind or an abandoned surgery), recurrence was only assigned if there was evidence that lesions had developed de novo in the successive surgery (for example, in a new location to that recorded in the previous report). Patients with only evidence of residual disease were not included in the recurrent endometriosis group. Recurrent endometriosis, record of two or more surgeries where endometriosis was visualised, was reported in *n* = 94. There were *n* = 288 participants grouped as having a single diagnosis of endometriosis (one endometriosis positive surgery only and no self-report of a previous endometriosis diagnosis), and *n* = 188 non-endometriosis controls. Note that non-endometriosis control and single diagnosis groups may have undergone more than one laparoscopy, but an endometriosis diagnosis was not confirmed (control), was only positive on one occasion (single diagnosis) or was ruled out as being de novo growth (residual disease).

Data from available ultrasound reports and positive or negative cervical intraepithelial neoplasia (CIN) findings on Papanicolaou test pathology screens were paired to the closest surgical date. Ultrasound and CIN findings were only available for participants who had these procedures undertaken at RWH.

### Statistical analysis

Analysis was undertaken using the R programming language [14]. For both analyses, groups were first analysed using univariate logistic regression for each variable separately. Numerical variables were presented as means and range, and binary and categorical variables were presented as *n* numbers and percentages, and as odds ratios (OR) with 95% confidence intervals (CI) with significance denoted by *p* values < 0.05. For both analyses, a multivariate logistic regression with least absolute shrinkage and selection operator (Lasso) regularisation was performed using the glmnet R package [15, 16], with test performance reported using receiver operating characteristic (ROC), sensitivity and specificity for each analysis. Variables with > 5% missing data were not included and variables with ≤ 5% missing values were imputed with either the median value for numeric variables or most frequent value for non-numeric variables. The purpose of the multivariate Lasso model was to guide feature selection for a prediction model. Lasso analysis limits the number of selected prognostic features remaining in a model by penalising the absolute values of coefficients, resulting in shrinking some coefficients to zero.

Ultrasound data was included in the surgically confirmed endometriosis analysis, when available. As smaller numbers were available, ultrasound data was analysed independently using univariate regression analysis only, and a Lasso regression and risk and survival analyses were not undertaken on these data.

For both analyses, Risk-calibrated Supersparse Linear Integer Models (RiskSLIM) were employed to create a simple scoring system associated with the probability of patients developing recurrent disease [17]. The RiskSLIM method uses optimisation techniques to find the best logistic regression model with a limited number of risk factors [18]. Variables with > 5% missing data were not included and the RiskSLIM model’s maximum number of factors was limited to 5. Evaluation of the RiskSLIM was performed by fivefold cross-validation (5-CV), where data was randomly split into 5 parts, fit using 4 of the 5 folds, and validated on the last fold. This was repeated 5 times to estimate the area under the curve (AUC) and risk calibration [18].

For the surgically confirmed endometriosis analysis, time gap between surgical procedures was used to conduct a survival analysis that determined the impact of variables on the risk of recurrent endometriosis versus a single diagnosis of endometriosis. Using the survival R package [19], Cox proportional hazards ratios (HR) (95% CI) were calculated to measure the effect of the hazard rate (adjusted *p* values < 0.05 were considered significant). Data was also presented as survival curves generated using the survminer R package [20], and fixed time-points of 2 and 5 years post index surgery were tabulated (Kaplan–Meier *p* values of < 0.05 considered significant). Multivariate Lasso analysis was employed to determine which combination of variables provided a good model for prediction.

### Role of the funding source

This work was partially supported by National Health and Medical Research Council (NHMRC) Grants GNT1105321 and GNT1012245 (P.A.W.R., G.W.M., J.E.G., S.J.H-C.), GNT1026033 (G.W.M.) and NHMRC Medical Research Future Fund GNT1199715 (P.A.W.R., M.H., S.J.H-C. and J.F.D.). G.W.M. was supported by NHMRC Fellowships GNT1078399 and GNT1177194. NHMRC played no role in the study design; in the collection, analysis and interpretation of data; in the writing of the report; or in the decision to submit the paper for publication.

## Results

Table 1 presents the results for the analysis of self-reported endometriosis recurrence. Tables 2 and 3 present the results for the surgically confirmed endometriosis recurrence analysis.

**Table 1:** Univariate and multivariate logistic regression results for the self-reported recurrent endometriosis analysis

	**Non-endometriosis control**	**Single endometriosis diagnosis**	**Recurrent endometriosis**	**Non-endometriosis control vs recurrent endometriosis**			**Non-endometriosis control vs single endometriosis diagnosis**			**Single endometriosis diagnosis vs recurrent endometriosis**		
				Univariate analysis		Multivariate Lasso	Univariate analysis		Multivariate Lasso	Univariate analysis		Multivariate Lasso
				OR, 95% CI	*p* value	Coef	OR (95% CI)	*p* value	Coef	OR (95% CI)	*p* value	Coef
**Age (years)**												
*n*	188	282	221									
Mean (range)	29.05 (18–50)	29.17 (18–47)	31.79 (18–47)		< 0.001			0.862			< 0.001	
18–24 (*n*, %)	63 (33.5)	73 (25.9)	38 (17.2)	–			–			–		
25–29 (*n*, %)	45 (23.9)	94 (33.3)	55 (24.9)	2.03 (1.15–3.56)	0.014	0.000	1.80 (1.10–2.94)	0.018	0.270	1.12 (0.67–1.88)	0.656	0.000
30–34 (*n*, %)	39 (20.7)	56 (19.9)	46 (20.8)	1.96 (1.09–3.52)	0.025	0.000	1.24 (0.73–2.10)	0.428	0.000	1.58 (0.91–2.74)	0.106	0.000
35+ (*n*, %)	41 (21.8)	59 (20.9)	82 (37.1)	3.32 (1.91–5.75)	< 0.001	0.641	1.24 (0.74–2.09)	0.416	0.484	2.67 (1.59–4.47)	< 0.001	0.223
**Past smoker**^**a**^												
No (*n*, %)	111 (59.0)	179 (63.5)	127 (57.5)	–						–		
Yes (*n*, %)	77 (41.0)	103 (36.5)	94 (42.5)	1.07 (0.72–1.58)	0.747	0.000	0.83 (0.57–1.21)	0.333	0.000	1.29 (0.90–1.84)	0.171	0.000
**Current smoker**^**a**^												
No (*n*, %)	139 (73.9)	219 (77.7)	160 (72.4)	–						–		
Yes (*n*, %)	49 (26.1)	63 (22.3)	61 (27.6)	1.08 (0.70–1.68)	0.727	0.000	0.82 (0.53–1.25)	0.354	−0.153	1.33 (0.88–1.99)	0.175	0.230
**Never smoked**^**a**^												
No (*n*, %)	78 (41.5)	103 (36.5)	94 (42.5)	–						–		
Yes (*n*, %)	110 (58.5)	179 (63.5)	127 (57.5)	0.96 (0.65–1.42)	0.831	0.000	1.23 (0.84–1.80)	0.279	0.000	0.78 (0.54–1.11)	0.171	0.000
**Age of menarche (years)**^**a**^												
*n*	188	281	221									
Mean (range)	12.57 (8–16)	12.93 (8–19)	12.50 (8–17)		0.655			0.025			0.006	
Under 12 (*n*, %)	47 (25.0)	53 (18.9)	63 (28.5)	–			–			–		
12–14 (*n*, %)	121 (64.4)	182 (64.8)	134 (60.6)	0.83 (0.53–1.30)	0.406	0.000	1.33 (0.85–2.10)	0.215	0.000	0.62 (0.40–0.95)	0.028	−0.116
15+ (*n*, %)	20 (10.6)	46 (16.4)	24 (10.9)	0.90 (0.44–1.81)	0.758	0.000	2.04 (1.06–3.93)	0.033	0.150	0.44 (0.24–0.81)	0.009	−0.302
**Gravidity**^**a**^												
*n*	188	281	221									
Mean (range)	1.12 (0–11)	0.42 (0–5)	0.80 (0–6)		0.046			< 0.001			< 0.001	
0	115 (61.2)	208 (74.0)	135 (61.1)	–			–			–		
1–2	37 (19.7)	65 (23.1)	62 (28.1)	1.43 (0.89–2.30)	0.144	0.000	0.97 (0.61–1.54)	0.902	0.000	1.47 (0.98–2.21)	0.066	0.000
3+	36 (19.1)	8 (2.8)	24 (10.9)	0.57 (0.32–1.01)	0.053	−0.530	0.12 (0.06–0.27)	< 0.001	−1.797	4.62 (2.02–10.59)	< 0.001	0.658
**Parity**^**a**^												
*n*	188	281	221									
Mean (range)	0.56 (0–5)	0.17 (0–4)	0.47 (0–4)		0.298			< 0.001			< 0.001	
0	135 (71.8)	251 (89.3)	157 (71.0)	–			–			–		
1–2	39 (20.7)	27 (9.6)	58 (26.2)	1.28 (0.80–2.04)	0.302	0.000	0.37 (0.22–0.63)	< 0.001	−0.519	3.43 (2.09–5.65)	<0.001	0.629
3+	14 (7.4)	3 (1.1)	6 (2.7)	0.37 (0.14–0.99)	0.047	−0.423	0.12 (0.03–0.41)	0.001	−0.115	3.20 (0.79–12.97)	0.104	0.000
**Systolic blood pressure (mmHg)**												
*n*	186	282	221									
Mean (range)	116.20 (89–159)	114.60 (82–158)	117.5 (85–152)		0.344			0.192			0.015	
Under 120	115 (61.8)	182 (64.5)	131 (59.3)	–			–			–		
120–129	40 (21.5)	69 (24.5)	42 (19.0)	0.92 (0.56–1.52)	0.750	0.000	1.09 (0.69–1.72)	0.710	0.115	0.85 (0.54–1.32)	0.460	−0.115
130–139	20 (10.8)	19 (6.7)	32 (14.5)	1.40 (0.76–2.59)	0.277	0.000	0.60 (0.31–1.17)	0.135	−0.165	2.34 (1.27–4.31)	0.006	0.480
140+	11 (5.9)	12 (4.3)	16 (7.2)	1.28 (0.57–2.86)	0.553	0.000	0.69 (0.29–1.61)	0.391	0.000	1.85 (0.85–4.05)	0.122	0.174
**Diastolic blood pressure (mmHg)**												
*n*	186	282	221									
Mean (range)	73.16 (50–100)	73.16 (48–106)	74.72 (43–101)		0.127			1.000			0.083	
Under 80	131 (70.4)	208 (73.8)	146 (66.1)				–					
80–84	33 (17.7)	44 (15.6)	33 (14.9)	0.90 (0.52–1.54)	0.692	0.000	0.84 (0.51–1.39)	0.495	0.000	1.07 (0.65–1.76)	0.794	−0.047
85–89	13 (7.0)	15 (5.3)	20 (9.0)	1.38 (0.66–2.88)	0.391	0.000	0.73 (0.34–1.58)	0.419	0.000	1.90 (0.94–3.83)	0.073	0.000
90+	9 (4.8)	15 (5.3)	22 (10.0)	2.19 (0.98–4.93)	0.058	0.126	1.05 (0.45–2.47)	0.912	0.133	2.09 (1.05–4.16)	0.036	0.061
**BMI (kg/m**^**2**^**)**												
*n*	188	282	221									
Mean (range)	26.71 (16.05–49.02)	24.56 (15.43–47.9)	25.35 (17.23–50.44)		0.018			< 0.001			0.099	
Normal (18.5–24.9 kg/m^2^)	87 (46.3)	170 (60.3)	124 (56.1)	–			–					
Underweight (< 18.5 kg/m^2^)	2 (1.1)	14 (5.0)	8 (3.6)	2.81 (0.58–13.54)	0.199	0.000	3.58 (0.80–16.12)	0.096	0.670	0.78 (0.32–1.92)	0.595	−0.415
Pre-obese (25–29.9 kg/m^2^)	49 (26.1)	64 (22.7)	55 (24.9)	0.79 (0.49–1.26)	0.322	0.000	0.67 (0.42–1.05)	0.081	−0.140	1.18 (0.77–1.81)	0.453	0.000
Obese (≥ 30 kg/m^2^)	50 (26.6)	34 (12.1)	34 (15.4)	0.48 (0.29–0.80)	0.005	−0.542	0.35 (0.21–0.58)	< 0.001	−0.956	1.37 (0.81–2.33)	0.242	0.000
**Severe menstrual pain**^**a**^												
No (*n*, %)	11 (5.9)	14 (5.0)	13 (5.9)							–		
Yes (*n*, %)	177 (94.1)	268 (95.0)	208 (94.1)	0.99 (0.43–2.27)	0.989	0.000	1.19 (0.53–2.68)	0.675	0.000	0.84 (0.38–1.82)	0.651	−0.100
**Severe pelvic pain (non-menstrual)**^**a**^												
No (*n*, %)	28 (14.9)	56 (19.9)	34 (15.4)							–		
Yes (*n*, %)	160 (85.1)	226 (80.1)	187 (84.6)	0.96 (0.56–1.66)	0.890	0.000	0.71 (0.43–1.16)	0.170	−0.176	1.36 (0.85–2.18)	0.195	0.013
**Dyspareunia**^**a**^												
No (*n*, %)	38 (20.2)	72 (25.5)	47 (21.3)							–		
Yes (*n*, %)	150 (79.8)	210 (74.5)	174 (78.7)	0.94 (0.58–1.52)	0.793	0.000	0.74 (0.47–1.15)	0.183	−0.101	1.27 (0.83–1.93)	0.265	0.014
**Attendance at an emergency room for menstrual/pelvic pain**^**a**^												
No (*n*, %)	131 (69.7)	172 (61.0)	95 (43.0)							–		
Yes (*n*, %)	57 (30.3)	110 (39.0)	126 (57.0)	3.05 (2.02–4.59)	< 0.001	0.872	1.47 (0.99–2.18)	0.054	0.463	2.07 (1.45–2.97)	< 0.001	0.574
**Currently taking hormone medication**^**a**^												
No (*n*, %)	95 (50.5)	178 (63.1)	134 (60.6)							–		
Yes (*n*, %)	93 (49.5)	104 (36.9)	87 (39.4)	0.66 (0.45–0.98)	0.041	−0.216	0.60 (0.41–0.87)	0.007	−0.543	1.11 (0.77–1.60)	0.568	0.183
**Previous hysterectomy**^**a**^												
No (*n*, %)	187 (99.5)	282 (100.0)	216 (97.7)									
Yes (*n*, %)	1 (0.5)	0 (0.0)	5 (2.3)	4.33 (0.50–37.38)	0.183	0.000		N/A	N/A		N/A	N/A
**Ovarian cysts**^**a**^												
No (*n*, %)	108 (57.4)	147 (52.1)	86 (38.9)							–		
Yes (*n*, %)	80 (42.7)	135 (47.9)	135 (61.1)	2.12 (1.43–3.15)	< 0.001	0.320	1.24 (0.85–1.80)	0.257	0.000	1.71 (1.20–2.44)	0.003	0.000
**Fibrocystic breasts**^**a**^												
No (*n*, %)	176 (93.6)	269 (95.4)	206 (93.2)							–		
Yes (*n*, %)	12 (6.4)	13 (4.6)	15 (6.8)	1.07 (0.49–2.34)	0.870	0.000	0.71 (0.32–1.59)	0.403	−0.505	1.51 (0.70–3.24)	0.293	0.000
**Uterine fibroids**^**a**^												
No (*n*, %)	169 (89.9)	270 (95.7)	191 (86.4)							–		
Yes (*n*, %)	19 (10.1)	12 (4.3)	30 (13.6)	1.40 (0.76–2.57)	0.283	0.000	0.40 (0.19–0.84)	0.015	−0.511	3.53 (1.76–7.08)	<0.001	0.524
**Polycystic ovary disease**^**a**^												
No (*n*, %)	148 (78.7)	230 (81.6)	177 (80.1)							–		
Yes (*n*, %)	40 (21.3)	52 (18.4)	44 (19.9)	0.92 (0.57–1.49)	0.733	0.000	0.84 (0.53–1.33)	0.448	0.000	1.10 (0.70–1.72)	0.677	0.000
**Adenomyosis**^**a**^												
No (*n*, %)	181 (96.3)	269 (95.4)	181 (81.9)							–		
Yes (*n*, %)	7 (3.7)	13 (4.6)	40 (18.1)	5.71 (2.49–13.09)	< 0.001	0.908	1.25 (0.49–3.19)	0.642	0.246	4.57 (2.38–8.79)	< 0.001	0.759
**Pre-cancer of the cervix or an abnormal Papanicolaou test**^**a**^												
No (*n*, %)	180 (95.7)	268 (95.0)	208 (94.1)							–		
Yes (*n*, %)	8 (4.3)	14 (5.0)	13 (5.9)	1.41 (0.57–3.47)	0.459	0.000	1.18 (0.48–2.86)	0.722	0.000	1.20 (0.55–2.60)	0.651	0.000
**Food allergies or intolerances**^**a**^												
No (*n*, %)	143 (76.1)	216 (76.6)	155 (70.1)							–		
Yes (*n*, %)	45 (23.9)	66 (23.4)	66 (29.9)	1.35 (0.87–2.10)	0.180	0.000	0.97 (0.63–1.50)	0.894	0.000	1.39 (0.94–2.08)	0.103	0.313
**Disturbance to taste or smell**^**a**^												
No (*n*, %)	173 (92.0)	267 (94.7)	195 (88.2)							–		
Yes (*n*, %)	15 (8.0)	15 (5.3)	26 (11.8)	1.54 (0.79–3.00)	0.207	0.000	0.65 (0.31–1.36)	0.251	0.000	2.37 (1.22–4.60)	0.010	0.173
**Hay fever**^**a**^												
No (*n*, %)	106 (56.4)	176 (62.4)	116 (52.5)							–		
Yes (*n*, %)	82 (43.6)	106 (37.6)	105 (47.5)	1.17 (0.79–1.73)	0.431	0.000	0.78 (0.53–1.13)	0.192	−0.266	1.50 (1.05–2.15)	0.025	0.376
**Eczema**^**a**^												
No (*n*, %)	146 (77.7)	192 (68.1)	174 (78.7)							–		
Yes (*n*, %)	42 (22.3)	90 (31.9)	47 (21.3)	0.94 (0.59–1.50)	0.793	0.000	1.63 (1.07–2.49)	0.024	0.340	0.58 (0.38–0.87)	0.008	−0.565
**Previous diagnosis of endometriosis**^**a**^												
No (*n*, %)	188 (100.0)	282 (100.0)	0 (0.0)									
Yes (*n*, %)	0 (0.0)	0 (0.0)	221 (100.0)		N/A	N/A		N/A	N/A		N/A	N/A
**Family history of endometriosis**^**a**^												
No (*n*, %)	138 (74.2)	211 (74.8)	149 (67.4)							–		
Yes (*n*, %)	48 (25.8)	70 (24.8)	72 (32.6)	1.39 (0.90–2.14)	0.136	0.047	0.95 (0.62–1.46)	0.827	0.000	1.46 (0.99–2.15)	0.059	0.369
**rASRM score**												
*n*		282	221									
Mean (range)	N/A	14.6 (1–134)	27.39 (1–142)		N/A			N/A			< 0.001	0.000
**Stage of endometriosis (rASRM)**												
Stage 1 (*n*, %)		175 (62.1)	88 (39.8)							–		
Stage 2 (*n*, %)		43 (15.2)	34 (15.4)							1.57 (0.94–2.64)	0.087	0.000
Stage 3 (*n*, %)		30 (10.6)	35 (15.8)							2.32 (1.34–4.02)	0.003	0.033
Stage 4 (*n*, %)	N/A	34 (12.1)	64 (29.0)		N/A			N/A		3.74 (2.30–6.10)	< 0.001	0.278
**Adhesions**												
No (*n*, %)		189 (67.0)	90 (40.7)									
Yes (*n*, %)	N/A	93 (33.0)	131 (59.3)		N/A			N/A		2.96 (2.05–4.26)	< 0.001	0.637
**Superficial ovarian lesion(s)**												
No (*n*, %)		245 (86.9)	198 (89.6)									
Yes (*n*, %)	N/A	37 (13.1)	23 (10.4)		N/A			N/A		0.77 (0.44–1.34)	0.352	0.000
**Deep ovarian lesion(s)**												
No (*n*, %)		219 (77.7)	141 (63.8)									
Yes (*n*, %)	N/A	63 (22.3)	80 (36.2)		N/A			N/A		1.97 (1.33–2.92)	0.001	0.000
**Superficial peritoneal lesion(s)**												
No (*n*, %)		32 (11.3)	48 (21.7)									
Yes (*n*, %)	N/A	250 (88.7)	173 (78.3)		N/A			N/A		0.46 (0.28–0.75)	0.002	−0.002
**Deep peritoneal lesion(s)**												
No (*n*, %)		203 (72.0)	125 (56.6)									
Yes (*n*, %)	N/A	79 (28.0)	96 (43.4)		N/A			N/A		1.97 (1.36–2.86)	< 0.001	0.290
**Pouch of Douglas**												
No (*n*, %)		123 (43.6)	99 (44.8)									
Yes (*n*, %)	N/A	159 (56.4)	122 (55.2)		N/A			N/A		0.95 (0.67–1.36)	0.792	0.000
**UV pouch**												
No (*n*, %)		210 (74.5)	167 (75.6)									
Yes (*n*, %)	N/A	72 (25.5)	54 (24.4)		N/A			N/A		0.94 (0.63–1.42)	0.778	0.000
**Bladder**												
No (*n*, %)		281 (99.6)	216 (97.7)									
Yes (*n*, %)	N/A	1 (0.4)	5 (2.3)		N/A			N/A		6.50 (0.75–56.08)	0.088	0.716
**Bowel**												
No (*n*, %)		250 (88.7)	176 (79.6)									
Yes (*n*, %)	N/A	32 (11.3)	45 (20.4)		N/A			N/A		2.00 (1.22–3.27)	0.006	0.000
**Tube**												
No (*n*, %)		267 (94.7)	209 (94.6)									
Yes (*n*, %)	N/A	15 (5.3)	12 (5.4)		N/A			N/A		1.02 (0.47–2.23)	0.956	−0.109
**Pelvic side wall**												
No (*n*, %)		90 (31.9)	73 (33.0)									
Yes (*n*, %)	N/A	192 (68.1)	148 (67.0)		N/A			N/A		0.95 (0.65–1.38)	0.791	0.000
**Pararectal space**												
No (*n*, %)		239 (84.8)	203 (91.9)									
Yes (*n*, %)	N/A	43 (15.2)	18 (8.1)		N/A			N/A		0.49 (0.28–0.88)	0.017	−0.547
**Uterosacral ligament**												
No (*n*, %)		151 (53.5)	123 (55.7)									
Yes (*n*, %)	N/A	131 (46.5)	98 (44.3)		N/A			N/A		0.92 (0.64–1.31)	0.637	0.000
**Other**												
No (*n*, %)		209 (74.1)	146 (66.1)									
Yes (*n*, %)	N/A	73 (25.9)	75 (33.9)		N/A			N/A		1.47 (1.00–2.16)	0.050	0.000

^a^Patient reported variable

**Table 2:** Univariate and multivariate logistic regression results for the surgically confirmed recurrent endometriosis analysis

	**Non-endometriosis control**	**Single endometriosis diagnosis**	**Recurrent endometriosis**	**Non-endometriosis control vs recurrent endometriosis**			**Non-endometriosis control vs single endometriosis diagnosis**			**Single endometriosis diagnosis vs recurrent endometriosis**		
				Univariate analysis		Multivariate Lasso	Univariate analysis		Multivariate Lasso	Univariate analysis		Multivariate Lasso
				OR (95% CI)	*p* value	Coef	OR (95% CI)	*p* value	Coef	OR (95% CI)	*p* value	Coef
**Age (years)**												
*n*	188	288	94									
Mean (range)	29.05 (18–50)	29.17 (18–47)	31.26 (18–45)		0.017			0.854			0.008	
18–24 (*n*, %)	63 (33.5)	76 (26.4)	17 (18.1)	–			–			–		
25–29 (*n*, %)	45 (23.9)	95 (33.0)	22 (23.4)	1.81 (0.86–3.80)	0.115	0.000	1.75 (1.08–2.85)	0.024	0.219	1.04 (0.51–2.09)	0.923	−0.027
30–34 (*n*, %)	39 (20.7)	56 (19.4)	25 (26.6)	2.38 (1.14–4.95)	0.021	0.000	1.19 (0.70–2.02)	0.518	0.000	2.00 (0.98–4.04)	0.055	0.000
35+ (*n*, %)	41 (21.8)	61 (21.2)	30 (31.9)	2.71 (1.33–5.53)	0.006	0.131	1.23 (0.73–2.07)	0.427	0.353	2.20 (1.11–4.36)	0.024	0.000
**Past smoker**^**a**^												
No (*n*, %)	111 (59.0)	181 (62.8)	46 (48.9)									
Yes (*n*, %)	77 (41.0)	107 (37.2)	48 (51.1)	1.50 (0.91–2.48)	0.108	0.000	0.85 (0.58–1.24)	0.405	0.000	1.77 (1.10–2.82)	0.018	0.317
**Current smoker**^**a**^												
No (*n*, %)	139 (73.9)	221 (76.6)	66 (70.2)									
Yes (*n*, %)	49 (26.1)	67 (23.3)	28 (29.8)	1.20 (0.69–2.08)	0.509	0.000	0.86 (0.56–1.32)	0.487	−0.066	1.40 (0.83–2.35)	0.205	0.000
**Never smoked**^**a**^												
No (*n*, %)	78 (41.5)	107 (37.2)	48 (51.1)									
Yes (*n*, %)	110 (58.5)	181 (62.8)	46 (48.9)	0.68 (0.41–1.12)	0.128	0.000	1.20 (0.82–1.75)	0.343	0.019	0.57 (0.35–0.91)	0.018	0.000
**Age of menarche (years)**^**a**^												
*n*	188	287	94									
Mean (range)	12.57 (8–16)	12.92 (8–19)	12.46 (9–17)		0.554			0.030			0.025	
Under 12 (*n*, %)	47 (25.0)	56 (19.5)	24 (25.5)	–			–			–		
12–14 (*n*, %)	121 (64.4)	183 (63.8)	63 (67.0)	1.02 (0.57–1.82)	0.947	0.000	1.27 (0.81–1.99)	0.300	0.000	0.80 (0.46–1.40)	0.441	0.000
15+ (*n*, %)	20 (10.6)	48 (16.7)	7 (7.4)	0.69 (0.25–1.85)	0.455	0.000	2.01 (1.05–3.86)	0.035	0.186	0.34 (0.13–0.86)	0.023	−0.435
**Gravidity**^**a**^												
*n*	188	287	94									
Mean (range)	1.12 (0–11)	0.43 (0–5)	0.78 (0–5)		0.109			< 0.001			0.003	
0	115 (61.2)	210 (73.2)	56 (59.6)	–			–			–		
1–2	37 (19.7)	69 (24.0)	29 (30.9)	1.61 (0.90–2.88)	0.109	0.000	1.02 (0.65–1.62)	0.929	0.000	1.58 (0.93–2.66)	0.089	0.000
3+	36 (19.1)	8 (2.8)	9 (9.6)	0.51 (0.23–1.14)	0.101	−0.366	0.12 (0.05–0.27)	< 0.001	−1.746	4.22 (1.56–11.43)	0.005	0.535
**Parity**^**a**^												
*n*	188	287	94									
Mean (range)	0.56 (0–5)	0.18 (0–4)	0.42 (0–3)		0.227			< 0.001			0.002	
0	135 (71.8)	255 (88.9)	68 (72.3)	–			–			–		
1–2	39 (20.7)	29 (10.1)	24 (25.5)	1.22 (0.68–2.20)	0.503	0.000	0.39 (0.23–0.66)	< 0.001	−0.485	3.10 (1.70–5.67)	< 0.001	0.345
3+	14 (7.4)	3 (1.0)	2 (2.1)	0.28 (0.06–1.28)	0.102	−0.048	0.11 (0.03–0.40)	0.001	0.000	2.50 (0.41–15.26)	0.321	0.000
**Systolic blood pressure (mmHg)**												
*n*	186	288	93									
Mean (range)	116.20 (89–159)	114.40 (82–158)	117.70 (90–152)		0.414			0.136			0.039	
Under 120	115 (61.8)	188 (65.3)	59 (63.4)	–			–			–		
120–129	40 (21.5)	69 (24.0)	13 (14.0)	0.63 (0.31–1.28)	0.201	0.000	1.06 (0.67–1.66)	0.816	0.076	0.60 (0.31–1.16)	0.130	−0.344
130–139	20 (10.8)	19 (6.6)	13 (14.0)	1.27 (0.59–2.72)	0.545	0.000	0.58 (0.30–1.14)	0.112	−0.154	2.18 (1.02–4.68)	0.045	0.003
140+	11 (5.9)	12 (4.2)	8 (8.6)	1.42 (0.54–3.71)	0.478	0.000	0.67 (0.29–1.56)	0.351	0.000	2.12 (0.83–5.44)	0.117	0.000
**Diastolic blood pressure (mmHg)**												
*n*	186	288	93									
Mean (range)	73.16 (50–100)	72.94 (48–106)	74.63 (43–100)		0.263			0.816			0.157	
Under 80	131 (70.4)	214 (74.3)	64 (68.8)	–						–		
80–84	33 (17.7)	44 (15.3)	12 (12.9)	0.74 (0.36–1.54)	0.425	0.000	0.82 (0.49–1.35)	0.427	0.000	0.91 (0.45–1.83)	0.795	0.000
85–89	13 (7.0)	15 (5.2)	5 (5.4)	0.79 (0.27–2.30)	0.662	0.000	0.71 (0.33–1.53)	0.379	0.000	1.11 (0.39–3.18)	0.840	0.000
90+	9 (4.8)	15 (5.2)	12 (12.9)	2.73 (1.09–6.81)	0.031	0.087	1.02 (0.43–2.40)	0.963	0.000	2.67 (1.19–6.01)	0.017	0.217
**BMI (kg/m**^**2**^**)**												
*n*	188	288	94									
Mean (range)	26.71 (16.05–49.02)	24.56 (15.43–47.90)	26.22 (17.23–50.44)		0.513			< 0.001			0.011	
Normal (18.5–24.9 kg/m^2^)	87 (46.3)	173 (60.1)	44 (46.8)	–			–					
Underweight (< 18.5 kg/m^2^)	2 (1.1)	14 (4.9)	5 (5.3)	4.94 (0.92–26.51)	0.062	0.000	3.52 (0.78–15.84)	0.101	0.539	1.40 (0.48–4.11)	0.535	0.000
Pre-obese (25–29.9 kg/m^2^)	49 (26.1)	66 (22.9)	22 (23.4)	0.89 (0.48–1.65)	0.707	0.000	0.68 (0.43–1.06)	0.090	−0.108	1.31 (0.73–2.35)	0.365	0.000
Obese (≥ 30 kg/m^2^)	50 (26.6)	35 (12.2)	23 (24.5)	0.91 (0.49–1.68)	0.762	0.000	0.35 (0.21–0.58)	< 0.001	−0.885	2.58 (1.39–4.81)	0.003	0.217
**Severe menstrual pain**^**a**^												
No (*n*, %)	11 (5.9)	20 (6.9)	5 (5.3)									
Yes (*n*, %)	177 (94.1)	268 (93.1)	89 (94.7)	1.11 (0.37–3.28)	0.856	0.000	0.83 (0.39–1.78)	0.637	0.000	1.33 (0.48–3.64)	0.581	0.000
**Severe pelvic pain (non-menstrual)**^**a**^												
No (*n*, %)	28 (14.9)	62 (21.5)	13 (13.8)									
Yes (*n*, %)	160 (85.1)	226 (78.5)	81 (86.2)	1.09 (0.54–2.22)	0.811	0.000	0.64 (0.39–1.04)	0.072	−0.190	1.71 (0.89–3.27)	0.106	0.107
**Dyspareunia**^**a**^												
No (*n*, %)	38 (20.2)	78 (27.1)	20 (21.3)									
Yes (*n*, %)	150 (79.8)	210 (72.9)	74 (78.7)	0.94 (0.51–1.72)	0.835	0.000	0.68 (0.44–1.06)	0.089	−0.122	1.37 (0.79–2.40)	0.264	0.000
**Attendance at an emergency room for menstrual/pelvic pain**^**a**^												
No (*n*, %)	131 (69.7)	178 (61.8)	45 (47.9)									
Yes (*n*, %)	57 (30.3)	110 (38.2)	49 (52.1)	2.50 (1.50–4.17)	< 0.001	0.523	1.42 (0.96–2.10)	0.079	0.366	1.76 (1.10–2.82)	0.018	0.170
**Currently taking hormone medication**^**a**^												
No (*n*, %)	95 (50.5)	181 (62.8)	62 (66.0)									
Yes (*n*, %)	93 (49.5)	107 (37.2)	32 (34.0)	0.53 (0.32–0.88)	0.015	−0.262	0.60 (0.42–0.88)	0.008	−0.482	0.87 (0.54–1.42)	0.586	0.000
**Previous hysterectomy**^**a**^												
No (*n*, %)	187 (99.5)	288 (100.0)	90 (95.7)									
Yes (*n*, %)	1 (0.5)	0 (0.0)	4 (4.3)	8.31 (0.92–75.44)	0.060	0.000		N/A			N/A	
**Ovarian cysts**^**a**^												
No (*n*, %)	108 (57.4)	153 (53.)	38 (40.4)									
Yes (*n*, %)	80 (42.7)	135 (46.9)	56 (59.6)	1.99 (1.20–3.29)	0.007	0.119	1.19 (0.82–1.73)	0.355	0.000	1.67 (1.04–2.68)	0.033	0.000
**Fibrocystic breasts**^**a**^												
No (*n*, %)	176 (93.6)	275 (95.5)	88 (93.6)									
Yes (*n*, %)	12 (6.4)	13 (4.5)	6 (6.4)	1.00 (0.36–2.75)	1.000	0.000	0.69 (0.31–1.55)	0.374	−0.414	1.44 (0.53–3.91)	0.471	0.000
**Uterine fibroids**^**a**^												
No (*n*, %)	169 (89.9)	276 (95.8)	82 (87.2)									
Yes (*n*, %)	19 (10.1)	12 (4.2)	12 (12.8)	1.30 (0.60–2.81)	0.502	0.000	0.39 (0.18–0.82)	0.013	−0.508	3.37 (1.46–7.78)	0.004	0.727
**Polycystic ovary disease**^**a**^												
No (*n*, %)	148 (78.7)	236 (81.9)	73 (77.7)									
Yes (*n*, %)	40 (21.3)	52 (18.1)	21 (22.3)	1.06 (0.59–1.94)	0.838	0.000	0.82 (0.51–1.29)	0.385	0.000	1.31 (0.74–2.31)	0.360	0.000
**Adenomyosis**^**a**^												
No (*n*, %)	181 (96.3)	275 (95.5)	74 (78.7)									
Yes (*n*, %)	7 (3.7)	13 (4.5)	20 (21.3)	6.99 (2.83–17.23)	< 0.001	1.356	1.22 (0.48–3.12)	0.675	0.217	5.72 (2.72–12.03)	< 0.001	1.250
**Pre-cancer of the cervix or an abnormal Papanicolaou test**^**a**^												
No (*n*, %)	180 (95.7)	274 (95.1)	85 (90.4)									
Yes (*n*, %)	8 (4.3)	14 (4.9)	9 (9.6)	2.38 (0.89–6.39)	0.085	0.000	1.15 (0.47–2.80)	0.758	0.000	2.07 (0.87–4.96)	0.102	0.339
**Cervical intraepithelial neoplasia (CIN) (pathology)**												
No (*n*, %)	171 (91.0)	259 (89.9)	77 (81.9)									
Yes (*n*, %)	17 (9.0)	29 (10.1)	17 (18.1)	2.22 (1.08–4.58)	0.031	0.184	1.13 (0.60–2.11)	0.711	0.000	1.97 (1.03–3.78)	0.041	0.456
**Food allergies or intolerances**^**a**^												
No (*n*, %)	143 (76.1)	222 (77.1)	66 (70.2)									
Yes (*n*, %)	45 (23.9)	66 (22.9)	28 (29.8)	1.35 (0.77–2.35)	0.291	0.000	0.94 (0.61–1.46)	0.797	0.000	1.43 (0.85–2.40)	0.181	0.127
**Disturbance to taste or smell**^**a**^												
No (*n*, %)	173 (92.0)	273 (94.8)	82 (87.2)									
Yes (*n*, %)	15 (8.0)	15 (5.2)	12 (12.8)	1.69 (0.76–3.77)	0.202	0.000	0.63 (0.30–1.33)	0.227	0.000	2.66 (1.20–5.92)	0.016	0.203
**Hay fever**^**a**^												
No (*n*, %)	106 (56.4)	182 (63.2)	45 (47.9)									
Yes (*n*, %)	82 (43.6)	106 (36.8)	49 (52.1)	1.41 (0.86–2.31)	0.177	0.199	0.75 (0.52–1.10)	0.138	−0.255	1.87 (1.17–2.99)	0.009	0.460
**Eczema**^**a**^												
No (*n*, %)	146 (77.7)	198 (68.8)	72 (76.6)									
Yes (*n*, %)	42 (22.3)	90 (31.3)	22 (23.4)	1.06 (0.59–1.91)	0.841	0.000	1.58 (1.03–2.41)	0.034	0.322	0.67 (0.39–1.15)	0.148	−0.279
**Previous diagnosis of endometriosis**^**a**^												
No (*n*, %)	188 (100.0)	288 (100.0)	19 (20.2)									
Yes (*n*, %)	0 (0.0)	0 (0.0)	75 (79.8)		N/A			N/A			N/A	
**Family history of endometriosis**^**a**^												
No (*n*, %)	138 (74.2)	215 (74.9)	64 (68.1)									
Yes (*n*, %)	48 (25.8)	72 (25.1)	30 (31.9)	1.35 (0.78–2.32)	0.282	0.000	0.96 (0.63–1.47)	0.861	0.000	1.40 (0.84–2.33)	0.196	0.145
**rASRM score**												
*n*		288	94									
Mean (range)	N/A	14.32 (1–134)	27.45 (1–142)		N/A			N/A			< 0.001	0.001
**Stage of endometriosis (rASRM)**												
Stage 1 (*n*, %)		181 (62.8)	36 (38.3)							–		
Stage 2 (*n*, %)		42 (14.9)	20 (21.3)							2.34 (1.23–4.43)	0.009	0.394
Stage 3 (*n*, %)	N/A	30 (10.4)	12 (12.8)							2.01 (0.94–4.30)	0.071	0.000
Stage 4 (*n*, %)		34 (11.8)	26 (27.7)		N/A			N/A		3.84 (2.06–7.17)	< 0.001	0.000
**Adhesions (surgical)**												
No (*n*, %)	150 (79.8)	191 (66.3)	36 (38.3)									
Yes (*n*, %)	38 (20.2)	97 (33.7)	58 (61.7)	6.36 (3.68–10.99)	< 0.001	1.524	2.00 (1.30–3.09)	0.002	0.408	3.17 (1.96–5.14)	< 0.001	0.715
**Superficial ovarian lesion(s)**												
No (*n*, %)		251 (87.2)	78 (83.0)									
Yes (*n*, %)	N/A	37 (12.8)	16 (17.0)		N/A			N/A		1.39 (0.73–2.64)	0.311	0.000
**Deep ovarian lesion(s)**												
No (*n*, %)		225 (78.1)	70 (74.5)									
Yes (*n*, %)	N/A	63 (21.9)	24 (25.5)		N/A			N/A		1.22 (0.71–2.10)	0.463	−0.192
**Superficial peritoneal lesion(s)**												
No (*n*, %)		32 (11.1)	19 (20.2)									
Yes (*n*, %)	N/A	256 (88.9)	75 (79.8)		N/A			N/A		0.49 (0.26–0.92)	0.026	0.000
**Deep peritoneal lesion(s)**												
No (*n*, %)		209 (72.6)	55 (58.5)									
Yes (*n*, %)	N/A	79 (27.4)	39 (41.5)		N/A			N/A		1.88 (1.15–3.05)	0.011	0.000
**Pouch of Douglas**												
No (*n*, %)		126 (43.8)	35 (37.2)									
Yes (*n*, %)	N/A	162 (56.3)	59 (62.8)		N/A			N/A		1.31 (0.81–2.12)	0.267	0.000
**UV pouch**												
No (*n*, %)		215 (74.7)	64 (68.1)									
Yes (*n*, %)	N/A	73 (25.3)	30 (31.9)		N/A			N/A		1.38 (0.83–2.30)	0.214	0.008
**Bladder**												
No (*n*, %)		287 (99.7)	92 (97.9)									
Yes (*n*, %)	N/A	1 (0.3)	2 (2.1)		N/A			N/A		6.24 (0.56–69.60)	0.137	0.699
**Bowel**												
No (*n*, %)		256 (88.9)	66 (70.2)									
Yes (*n*, %)	N/A	32 (11.1)	28 (29.8)		N/A			N/A		3.39 (1.91–6.03)	< 0.001	0.691
**Tubes**												
No (*n*, %)		273 (94.8)	86 (91.5)									
Yes (*n*, %)	N/A	15 (5.2)	8 (8.5)		N/A			N/A		1.69 (0.69–4.13)	0.247	0.000
**Pelvic side wall**												
No (*n*, %)		94 (32.6)	32 (34.0)									
Yes (*n*, %)	N/A	194 (67.4)	62 (66.0)		N/A			N/A		0.94 (0.57–1.54)	0.802	0.000
**Pararectal space**												
No (*n*, %)		245 (85.1)	87 (92.6)									
Yes (*n*, %)	N/A	43 (14.9)	7 (7.4)		N/A			N/A		0.46 (0.20–1.06)	0.067	−0.371
**Uterosacral ligament**												
No (*n*, %)		154 (53.5)	56 (59.6)									
Yes (*n*, %)	N/A	134 (46.5)	38 (40.4)		N/A			N/A		0.78 (0.49–1.25)	0.302	−0.074
**Other**												
No (*n*, %)		215 (74.7)	68 (72.3)									
Yes (*n*, %)	N/A	73 (25.3)	26 (27.7)		N/A			N/A		1.13 (0.67–1.90)	0.657	0.000

^a^Patient reported variable

**Table 3:** Univariate results for the surgically confirmed recurrent endometriosis analysis using ultrasound data

	**Non-endometriosis control**	**Single endometriosis diagnosis**	**Recurrent endometriosis**	**Non-endometriosis control vs recurrent endometriosis**		**Non-endometriosis control vs single endometriosis diagnosis**		**Single endometriosis diagnosis vs recurrent endometriosis**	
				Univariate analysis					
				OR, 95% CI	*p* value	OR, 95% CI	*p* value	OR, 95% CI	*p* value
**Uterine volume (ml)**									
*n*	67	105	63						
Mean (range)	69.70 (16.00–308.40)	67.00 (23.00–213.00)	85.81 (23.20–370.00)		0.119		0.672		0.017
Under 40 ml	16 (23.9)	14 (13.3)	5 (7.9)						
40–110 ml	44 (65.7)	82 (78.1)	45 (71.4)	3.27 (1.10–9.70)	0.033	2.13 (0.95–4.77)	0.066	1.54 (0.52–4.54)	0.437
> 110 ml	7 (10.4)	9 (8.6)	13 (20.6)	5.94 (1.52–23.18)	0.010	1.47 (0.43–4.98)	0.537	4.04 (1.07–15.27)	0.039
**Log uterine volume (ml)**									
*n*	67	105	63						
Mean (range)	1.77 (1.20–2.49)	1.79 (1.36–2.33)	1.87 (1.37–2.57)		0.023		0.460		0.021
**Uterine position**									
Anteverted	59 (90.8)	83 (79.8)	48 (77.4)					–	
Retroverted	6 (9.2)	20 (19.2)	12 (19.4)	2.46 (0.86–7.04)	0.094	2.37 (0.90–6.26)	0.082	1.04 (0.47–2.31)	0.928
Axial	0 (0.0)	1 (1.0)	2 (3.2)	N/A		N/A		3.46 (0.31–39.15)	0.316
**Fibroids present**									
No (*n*, %)	63 (88.7)	103 (88.8)	54 (79.4)						
Yes (*n*, %)	8 (11.3)	13 (11.2)	14 (20.6)	2.04 (0.80–5.24)	0.137	0.99 (0.39–2.53)	0.990	2.05 (0.90–4.68)	0.087
**No. of fibroids**									
*n*	71	116	68						
Mean (range)	0.23 (0–5)	0.13 (0–2)	0.34 (0–4)		0.421		0.294		0.034
**Volume of largest fibroid (ml)**									
*n*	6	11	11						
Mean (range)	58.62 (0.40–174.00)	2.89 (0.10–8.00)	27.74 (0.60–246.50)		0.414		0.148		0.188
**Ovarian cysts present**									
No (*n*, %)	54 (76.1)	77 (66.4)	41 (60.3)						
Yes (*n*, %)	17 (23.9)	39 (33.6)	27 (39.7)	2.09 (1.01–4.34)	0.048	1.61 (0.83–3.14)	0.163	1.30 (0.70–2.42)	0.407
**No. of ovarian cysts**									
*n*	71	116	68						
Mean (range)	0.34 (0–4)	0.67 (0–10)	0.62 (0–3)		0.047		0.078		0.773
**Polycystic ovaries present**									
No (*n*, %)	61 (85.9)	101 (87.1)	65 (95.6)						
Yes (*n*, %)	10 (14.1)	15 (12.9)	3 (4.4)	0.28 (0.07–1.07)	0.063	0.91 (0.38–2.14)	0.822	0.31 (0.09–1.12)	0.073
**Adenomyosis present**									
No (*n*, %)	66 (93.0)	92 (79.3)	51 (75.0)						
Yes (*n*, %)	5 (7.0)	24 (20.7)	17 (25.0)	4.40 (1.52–12.73)	0.006	3.44 (1.25–9.49)	0.017	1.28 (0.63–2.60)	0.498
**Adenomyosis linear striations present**									
No (*n*, %)	68 (95.8)	105 (90.5)	62 (91.2)						
Yes (*n*, %)	3 (4.2)	11 (9.5)	6 (8.8)	2.19 (0.53–9.15)	0.281	2.37 (0.64–8.82)	0.197	0.92 (0.33–2.62)	0.882
**Adenomyosis heterogeneous myometrium**									
No (*n*, %)	71 (100.0)	111 (95.7)	61 (89.7)						
Yes (*n*, %)	0 (0.0)	5 (4.3)	7 (10.3)	N/A		N/A		2.55 (0.78–8.37)	0.123
**Adenomyosis thickened posterior wall**									
No (*n*, %)	68 (95.8)	105 (90.5)	61 (89.7)						
Yes (*n*, %)	3 (4.2)	11 (9.5)	7 (10.3)	2.60 (0.64–10.51)	0.180	2.37 (0.64–8.82)	0.197	1.10 (0.40–2.97)	0.858
**Endometriosis present (including OMA)**									
No (*n*, %)	69 (97.2)	84 (72.4)	40 (58.8)						
Yes (*n*, %)	2 (2.8)	32 (27.6)	28 (41.2)	24.15 (5.46–106.78)	< 0.001	13.14 (3.04–56.80)	0.001	1.84 (0.98–3.46)	0.059
**Endometriosis present (excluding OMA)**									
No (*n*, %)	69 (97.2)	112 (96.6)	56 (82.4)						
Yes (*n*, %)	2 (2.8)	4 (3.4)	12 (17.6)	7.39 (1.59–34.41)	0.011	1.23 (0.22–6.91)	0.812	6.00 (1.85–19.45)	0.003
**Bilateral endometriomas**									
No (*n*, %)	71 (100.0)	107 (92.2)	63 (92.6)						
Yes (*n*, %)	0 (0.0)	9 (7.8)	5 (7.4)	N/A		N/A		0.94 (0.30–2.94)	0.920
**Adhesions (ultrasound)**									
No (*n*, %)	70 (98.6)	110 (94.8)	53 (77.9)						
Yes (*n*, %)	1 (1.4)	6 (5.2)	15 (22.1)	19.81 (2.54–154.74)	0.004	3.82 (0.45–32.39)	0.219	5.19 (1.91–14.13)	0.001

*OMA* Endometrioma

### Common features of endometriosis that were associated with recurrent disease

Several endometriosis-related features were repeatedly identified in association with recurrent disease. The recurrent endometriosis groups had increased odds of having adhesions visualised at the index surgery compared to the group with a single endometriosis diagnosis, in both the self-reported (Table 1) and surgically classified analysis (Table 2) (univariate OR 2.96 [95% CI 2.05–4.26] and OR 3.17 [1.96–5.14], both *p* values < 0.001). Adhesions detected at ultrasound prior to the index surgery were also significantly associated with recurrent disease compared to the single diagnosis group (univariate OR 5.19 [1.91–14.13], *p* value 0.001) and controls (univariate OR 19.81 [2.54–154.74], *p* value 0.004) (Table 3).

Higher rASRM scores were associated with recurrent disease versus a single diagnosis in both analyses (univariate *p* values < 0.001) (Tables 1 and 2). In the self-reported analysis, this was emphasised in association with stage 4 disease (OR 3.74 [2.30–6.10], *p* value < 0.001) and subsequently, the reported presence of deep peritoneal disease (OR 1.97 [1.36–2.86], *p* value < 0.001) (Table 1). Narrowing down, in the analysis using surgically classified recurrent disease, the presence of bowel lesions was significantly linked to recurrence (OR 3.39 [1.91–6.03], *p* value < 0.001) (Table 2). In contrast, patients with lesions in the pararectal space had a lower odds of recurrent endometriosis relative to having a single diagnosis of endometriosis (OR 0.49 [0.28–0.88], *p* value 0.017, and OR 0.46 [0.20–1.06], *p* value 0.067, respectively) (Tables 1 and 2).

### Other gynaecological features associated with recurrent endometriosis

Adenomyosis (patient reported), in both the self-reported and surgically confirmed analyses, was associated with an increased likelihood of endometriosis recurrence compared to a single diagnosis (Tables 1 and 2) (univariate OR 4.57 [2.38–8.79] and OR 5.72 [2.72–12.03], both *p* values < 0.001, respectively). Those with surgically confirmed recurrence had increased odds of adenomyosis as detected by ultrasound compared to controls (univariate OR 4.40 [1.52–12.73], *p* value 0.006) (Table 3). Also of note in Table 3, uterine volume (logged) was increased in the recurrent endometriosis group compared to controls and those with a single diagnosis (*p* value 0.023 and 0.021, respectively), which may be an indicator of adenomyosis.

Self-reported recurrent disease increased the odds for presentation at an ER compared to controls and those with a single diagnosis (both *p* values < 0.001) (Table 1). Similar outcomes were observed in the surgically confirmed analysis (Table 2), where recurrent endometriosis was associated with increased odds of presenting to an ER with pelvic pain (*p* values < 0.001 and 0.018). Dysmenorrhea, dyspareunia and severe (non-menstrual) pelvic pain were not significantly associated with endometriosis recurrence (Tables 1 and 2).

When comparing non-endometriosis controls to recurrent endometriosis cases, recurrent endometriosis was associated with reduced odds of taking hormone medication (Tables 1 and 2) (*p* value 0.041 and *p* value 0.015, respectively). However, this response was not limited to recurrent endometriosis, as those with a single endometriosis diagnosis were also found to have reduced odds of hormone medication usage compared to non-endometriosis controls (Tables 1 and 2) (*p* value 0.007 and *p* value 0.008, respectively).

Older age at the time of first menstrual period (in particular 15 + years) was associated with the single diagnosis of endometriosis group compared to the recurrence or control groups (Tables 1 and 2), albeit in opposite directions. Those with a single diagnosis of endometriosis had an increased odds of being 15 + years at the time of first menarche compared to non-endometriosis controls (Tables 1 and 2) (*p* value 0.033 and *p* value 0.035). However, the opposite effect was seen in the single diagnosis versus recurrent endometriosis groups, where older age at menarche (15 +) reduced the odds of recurrence relative to a single diagnosis of endometriosis (*p* value 0.009 and *p* value 0.023, respectively) (Tables 1 and 2).

Gravidity and parity were significantly associated with a single or recurrent endometriosis diagnosis (Tables 1 and 2). A similar pattern was observed for the single diagnosis group, which had reduced odds of reporting 3 + gravidity compared to controls (*p* value < 0.001 and *p* value < 0.001, respectively) (Tables 1 and 2), and reduced odds of reporting a parity between 1 and 2 or 3 + compared to controls (*p* values also ranging between < 0.001 and 0.001) (Tables 1 and 2). However, when comparing recurrent disease to a single diagnosis of endometriosis in both analytic groups (Tables 1 and 2), an increase of the odds of reporting a gravidity of 3 + (*p* value < 0.001 and 0.005, respectively) or a parity of 1–2 (*p* value, both < 0.001) was observed.

### Non-gynaecological factors associated with endometriosis recurrence

An obese BMI increased the likelihood of recurrence when compared to a single diagnosis of endometriosis (univariate OR 2.58 [1.39–4.81], *p* value 0.003) (Table 2). On the other hand, obesity was associated with lower odds of a single endometriosis diagnosis compared to non-endometriosis controls (both *p* values < 0.001) (Tables 1 and 2). In both the self-reported and surgically confirmed recurrence analyses, increased blood pressure was significant when recurrence was compared to a single diagnosis (univariate analysis) (Tables 1 and 2). In particular, higher systolic blood pressure (130–139 mmHg group) was associated with recurrent disease compared to a single endometriosis diagnosis (Tables 1 and 2) (*p* value 0.006 and *p* value 0.045, respectively). Overall, age was significantly related to disease recurrence; those with recurrent disease were older compared to non-endometriosis controls and those with a single diagnosis (Tables 1 and 2). In particular, in the self-reported endometriosis recurrence analysis, the recurrence group had increased odds of being in the older age group (35 + years) versus controls (*p* value < 0.001) and a single diagnosis (*p* value < 0.001) (Table 1). The same was true in the surgically confirmed group, with the 35 + age group significantly positively associated with recurrence when compared to non-endometriosis controls (*p* value 0.006) (Table 2).

### Predicting if endometriosis will reoccur

**Fig. 2:**
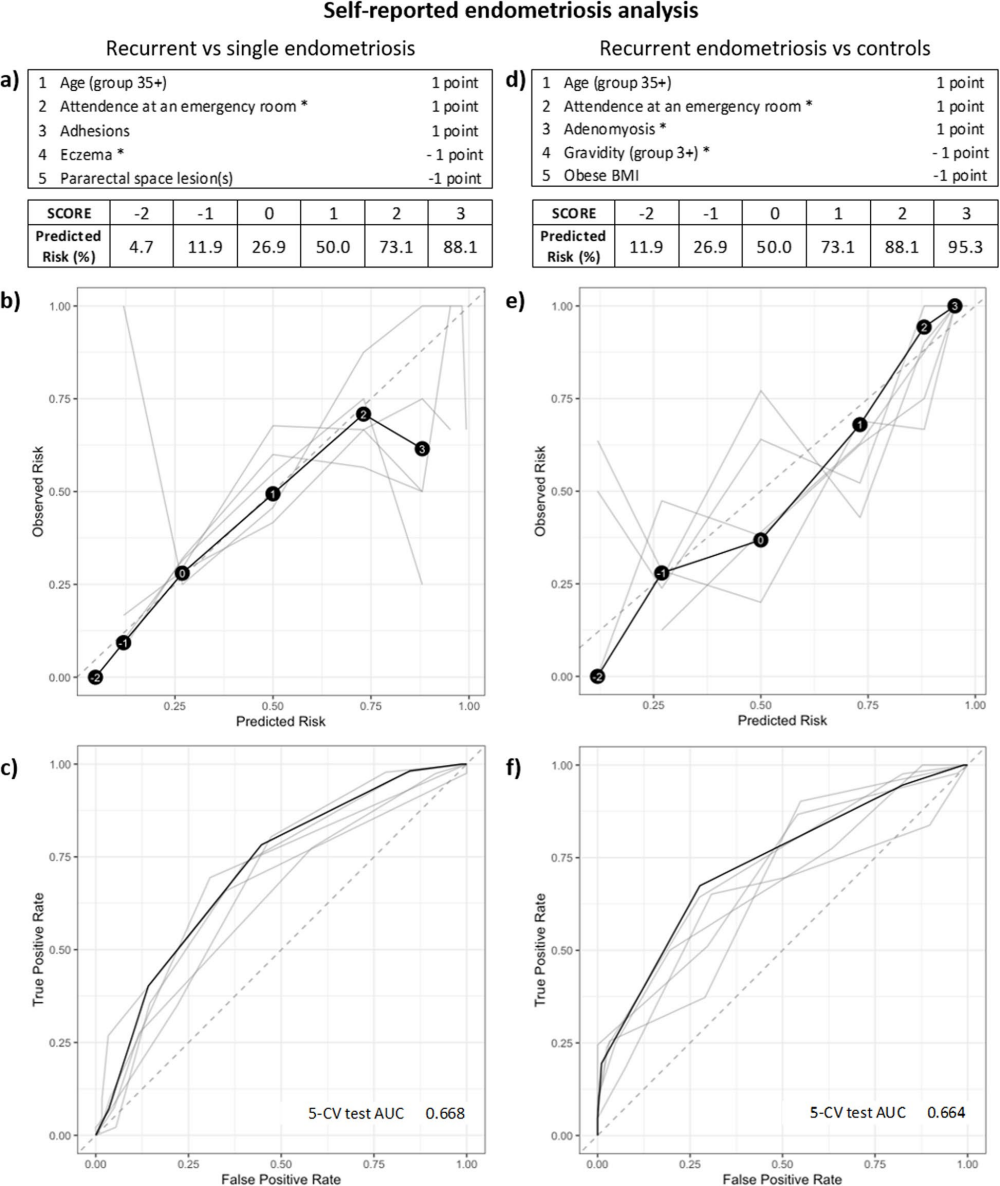
RiskSLIM scores to assess predicted risk of recurrent endometriosis in the self-reported endometriosis analysis group. **a** Tally of points and resulting scores for the various combinations of present features in the recurrent endometriosis versus single diagnosis of endometriosis comparison. Variables were selected to maximise the 5 cross-validation (CV) test AUC. The final score can sit between −2 and 3, with a predicted risk of recurrence at 4.7% for a score of −2 and 88.1% for a high score of 3. **d** Points and scores for the recurrent endometriosis versus non-endometriosis control comparison. The final score can also sit between −2 and 3, with a predicted risk of recurrence at 11.9% for a score of −2 and 95.3% for a high score of 3. **b** and **e** Calibration reliability graphs with observed risk (y-axis) and predicated risk (x-axis). The final model is shown in black (with risk scores in black circles), and the 5-CV models on test data shown in grey. The 45° dashed grey line represents a perfect risk calibration. **c** and **f** Receiver operating characteristic (ROC) curve with true positive rate (y-axis) and false positive rate (x-axis). The final model is shown in black and the 5-CV models on test data shown in grey. Area under the ROC curve (AUC) for the 5-CV test and the final model are illustrated on the bottom right of the ROC curve diagram

**Fig. 3:**
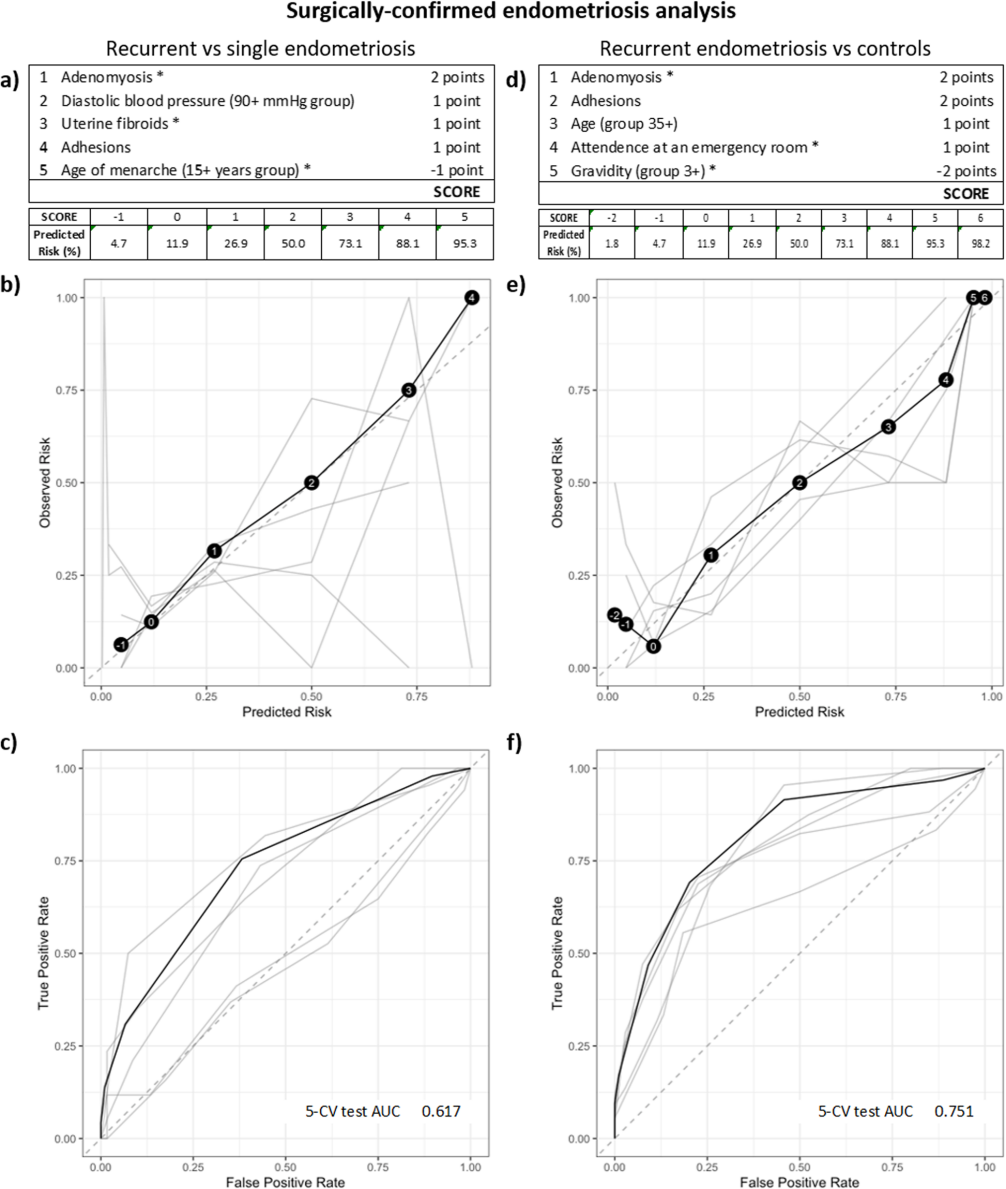
RiskSLIM scores to assess predicted risk of recurrent endometriosis in the surgically confirmed analysis group. **a** Tally of points and resulting scores for the various combinations of present features in the recurrent endometriosis versus single diagnosis of endometriosis comparison. Variables were selected to maximise the 5 cross-validation (CV) test AUC. The final score sat between −1 and 5, with a predicted risk of recurrence at 4.7% for a score of −1 and 95.3% for a high score of 5. **d** Points and scores for the recurrent endometriosis versus non-endometriosis control comparison. The final score sat between −2 and 6, with a predicted risk of recurrence at 1.8% for a score of −2 and 98.2% for a high score of 6. **b** and **e** Calibration reliability graphs with observed risk (y-axis) and predicated risk (x-axis). The final model is shown in black (with risk scores in black circles), and the 5-CV models on test data shown in grey. The 45° dashed grey line represents a perfect risk calibration. **c** and **f** Receiver operating characteristic (ROC) curve with true positive rate (y-axis) and false positive rate (x-axis). The final model is shown in black and the 5-CV models on test data shown in grey. Area under the ROC curve (AUC) for the 5-CV test and the final model are illustrated on the bottom right of the ROC curve diagram

RiskSLIM analyses, resulting in a points-based system to score the probability that patients will develop recurrent disease, were employed in both study groups. In the self-reported (Fig. 2a) and surgically confirmed analyses (Fig. 3a), adhesions were the one common variable that increased the prediction risk score in the models when comparing recurrent endometriosis to a single endometriosis diagnosis. When applying RiskSLIM to the non-endometriosis controls and recurrent endometriosis groups, adenomyosis was the common variable that increased the risk score in both groups, while higher gravidity (3 +) reduced the overall risk score in both groups (Figs. 2d and 3d). Using Fig. 3d as an example, adenomyosis (2 points), plus adhesions (2 points), plus age (35 +) (1 point), plus attendance at an ER (1 point) equals the highest possible RiskSLIM score of 6, which contributes to a 98.2% predicted risk for endometriosis recurrence (Fig. 3d, e). As demonstrated in Fig. 3f, this RiskSLIM model gave the highest AUC: a 5-CV AUC of 0.751. The more important outcome here is the prediction of recurrent disease compared to a single diagnosis, thus when using Fig. 3a as an example, adenomyosis (2 points), plus diastolic blood pressure (90 + mmHg) (1 point), plus uterine fibroids (1 point), plus adhesions (1 point) equals the highest possible RiskSLIM score of 5 for this model, which contributes to a 95.3% predicted risk for endometriosis recurrence (Fig. 3a, b). However, here we saw increased overall variability and low AUCs (5-CV AUC of 0.617, Fig. 3c and 5-CV AUC of 0.668, Fig. 2c). Unfortunately, the current models are not able to accurately discriminate for recurrent disease.

**Fig. 4:**
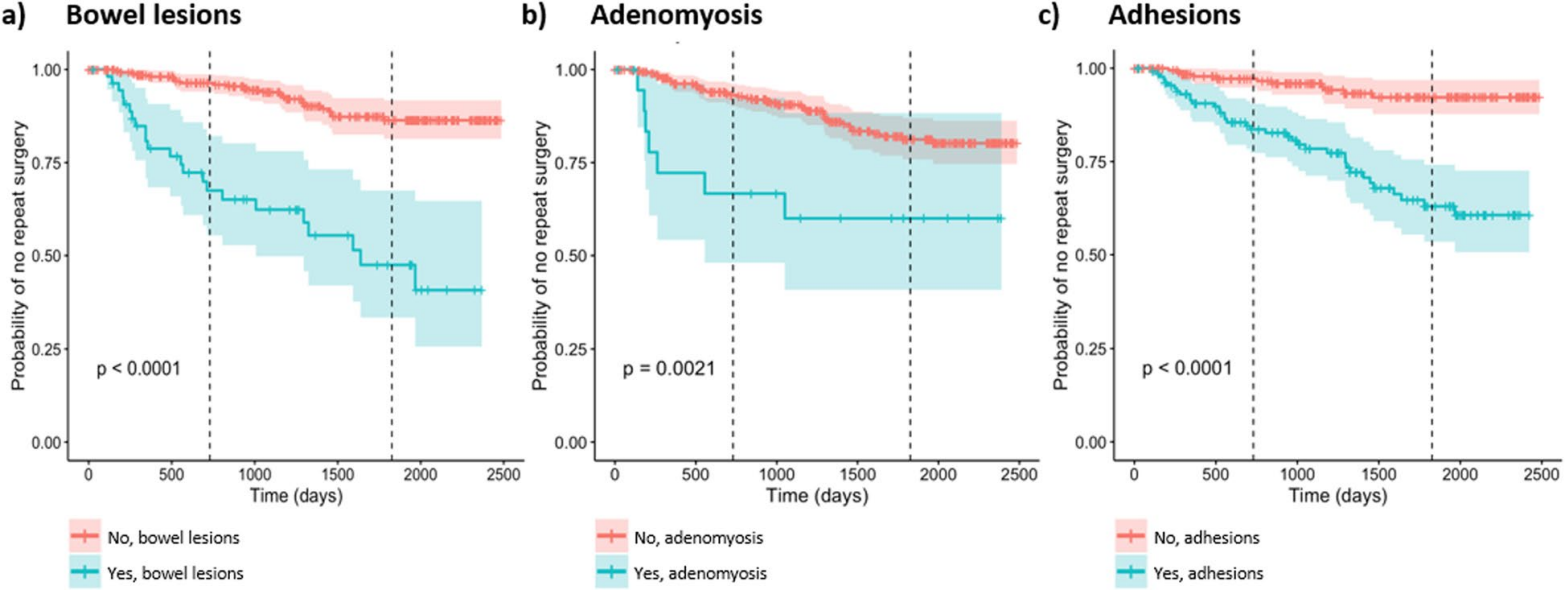
Kaplan-Meier curves for the surgically confirmed endometriosis recurrence group. Survival analysis was performed on data from patients with confirmed endometriosis (in the surgically confirmed analysis group). **a** Presence of lesions on the bowel, **b** self-reported diagnosis of adenomyosis and **c** presence of adhesions. **a**–**c** Red = no (variable not present), blue = yes (variable present). Vertical dashed line placed at 2 years and 5 years (time). Kaplan-Meier *p* values are included on the graph (bottom left)

Using dates of surgery, we were able to conduct a survival analysis on the surgically confirmed endometriosis group, allowing us to predict the probability of no disease recurrence. Self-reported previous diagnosis of endometriosis was a highly significant predictor of disease recurrence (univariate HR 32.46 [17.69–59.59], adj. *p* value < 0.001) (Table 4). For those who self-report a previous diagnosis, the 2- and 5-year probability of no recurrence was 38% and 3%, respectively, compared to 97% and 90% chance of no recurrence for those who did not report a previous diagnosis (Table 4 and Fig. 4a). Due to its strength, the feature ‘previous diagnosis of endometriosis’ was not included in the multivariate Lasso model.

**Table 4:** Cox proportional hazards regression and 2-year and 5-year probabilities from Kaplan-Meier estimations

**Factor**	***n***	**Univariate survival analysis**						**Multivariate**
		**Overall survival**			**Probability of no recurrence at 2 years**	**Probability of no recurrence at 5 years**	**Kaplan–Meier *p* value**	**Lasso coef**
		**HR (95% CI)**	***p* value**	**Adj. *p* value**				
**Age (years)**								
Overall	335	1.05 (1.01–1.09)	0.014	0.067				
18–24	84	–			0.97 (0.94–1.00)	0.86 (0.78–0.96)	0.049	
25–29	107	1.34 (0.55–3.29)	0.519	0.666	0.94 (0.89–0.99)	0.84 (0.75–0.93)		0.000
30–34	67	2.34 (0.96–5.73)	0.062	0.209	0.85 (0.77–0.95)	0.77 (0.66–0.90)		0.000
35 +	77	2.75 (1.18–6.44)	0.019	0.083	0.87 (0.79–0.96)	0.69 (0.57–0.85)		0.000
**Past smoker**^**a**^								
No	206	–			0.91 (0.86–0.95)	0.82 (0.76–0.89)	0.510	0.000
Yes	129	1.21 (0.69–2.14)	0.511	0.666	0.93 (0.89–0.98)	0.77 (0.69–0.87)		
**Current smoker**^**a**^								
No	258	–			0.91 (0.88–0.95)	0.79 (0.73–0.86)	0.600	0.000
Yes	77	0.83 (0.41–1.66)	0.597	0.736	0.93 (0.87–0.99)	0.82 (0.72–0.93)		
**Never smoked**^**a**^								
No	129	–			0.93 (0.89–0.98)	0.77 (0.69–0.87)	0.510	0.000
Yes	206	0.83 (0.47–1.46)	0.511	0.666	0.91 (0.86–0.95)	0.82 (0.76–0.89)		
**Age of menarche (years)**								
Overall	334	0.88 (0.74–1.05)	0.165	0.343				
Under 12	66	–			0.90 (0.83–0.98)	0.76 (0.63–0.90)	0.250	
12–14	217	0.90 (0.46–1.78)	0.760	0.836	0.91 (0.87–0.95)	0.79 (0.73–0.86)		0.000
15+	51	0.36 (0.10–1.29)	0.118	0.266	0.95 (0.89–1.00)	0.89 (0.75–1.00)		0.000
**Gravidity**^**a**^								
Overall	334	1.19 (0.91–1.57)	0.206	0.387				
0	242	–			0.92 (0.89–0.96)	0.82 (0.76–0.88)	0.110	
1–2	80	1.11 (0.57–2.16)	0.758	0.836	0.93 (0.86–0.99)	0.78 (0.68–0.90)		0.000
3+	12	2.89 (1.02–8.16)	0.046	0.170	0.67 (0.43–1.00)	0.56 (0.32–1.00)		0.000
**Parity**^**a**^								
Overall	334	1.35 (0.96–1.89)	0.087	0.237				
0	291	–			0.93 (0.90–0.96)	0.82 (0.77–0.88)	0.048	
1–2	38	2.01 (0.97–4.18)	0.060	0.209	0.84 (0.72–0.98)	0.67 (0.50–0.88)		0.000
3+	5	3.28 (0.79–13.63)	0.102	0.245	0.75 (0.43–1.00)	0.50 (0.19–1.00)		0.000
**Systolic blood pressure (mmHg)**								
Overall	334	1.00 (0.97–1.02)	0.753	0.836				
Under 120	220	–			0.90 (0.85–0.94)	0.79 (0.73–0.86)	0.700	
120–129	78	0.73 (0.35–1.52)	0.393	0.593	0.94 (0.89–1.00)	0.84 (0.75–0.94)		0.000
130–139	21	0.52 (0.12–2.17)	0.370	0.593	0.95 (0.86–1.00)	0.86 (0.70–1.00)		0.000
140+	15	0.97 (0.30–3.17)	0.960	0.960	1.00 (1.00–1.00)	0.75 (0.50–1.00)		0.000
**Diastolic blood pressure (mmHg)**								
Overall	334	1.00 (0.97–1.03)	0.797	0.864				
Under 80	250	–			0.91 (0.88–0.95)	0.79 (0.73–0.86)	0.160	
80–84	47	0.39 (0.12–1.26)	0.116	0.266	0.95 (0.88–1.00)	0.91 (0.83–1.00)		0.000
85–89	16	0.49 (0.07–3.55)	0.477	0.644	0.93 (0.80–1.00)	0.93 (0.80–1.00)		0.000
9+	21	1.66 (0.70–3.94)	0.250	0.437	0.85 (0.71–1.00)	0.66 (0.45–0.98)		0.000
**BMI (kg/m**^**2**^**)**								
Overall	335	1.03 (0.99–1.08)	0.178	0.351				
Normal (18.5–24.9)	194	–			0.95 (0.91–0.98)	0.82 (0.76–0.90)	0.011	
Underweight (< 18.5)	19	2.47 (0.93–6.52)	0.069	0.212	0.78 (0.61–1.00)	0.70 (0.51–0.97)		0.000
Pre-obese (25–29.9)	75	1.05 (0.48–2.29)	0.898	0.922	0.90 (0.83–0.97)	0.85 (0.76–0.95)		0.000
Obese (≥ 30)	47	2.75 (1.36–5.57)	0.005	0.034	0.88 (0.78–0.98)	0.65 (0.50–0.85)		0.000
**Severe menstrual pain**^**a**^								
No	22	–			0.88 (0.74–1.00)	0.88 (0.74–1.00)	0.470	0.000
Yes	313	1.67 (0.41–6.90)	0.476	0.644	0.92 (0.89–0.95)	0.79 (0.74–0.85)		
**Severe pelvic pain (non-menstrual)**^**a**^								
No	72	–			0.89 (0.82–0.97)	0.83 (0.74–0.94)	0.640	0.000
Yes	263	1.18 (0.59–2.37)	0.642	0.772	0.92 (0.89–0.96)	0.79 (0.73–0.86)		
**Dyspareunia**^**a**^								
No	91	–			0.90 (0.84–0.97)	0.83 (0.74–0.93)	0.850	0.000
Yes	244	0.94 (0.50–1.78)	0.855	0.901	0.92 (0.89–0.96)	0.79 (0.73–0.86)		
**Attendance at an emergency room for menstrual/pelvic pain**^**a**^								
No	204	–			0.94 (0.90–0.97)	0.82 (0.75–0.89)	0.300	0.000
Yes	131	1.35 (0.76–2.38)	0.307	0.507	0.88 (0.82–0.95)	0.77 (0.69–0.87)		
**Currently taking hormone medication**^**a**^								
No	216	–			0.91 (0.87–0.95)	0.76 (0.69–0.84)	0.062	0.000
Yes	119	0.54 (0.28–1.04)	0.066	0.212	0.93 (0.88–0.98)	0.87 (0.80–0.94)		
**Ovarian cysts**^**a**^								
No	175	–			0.92 (0.88–0.97)	0.82 (0.75–0.89)	0.460	0.000
Yes	160	1.24 (0.70–2.19)	0.460	0.644	0.91 (0.86–0.96)	0.78 (0.70–0.86)		
**Fibrocystic breasts**^**a**^								
No	319	–			0.92 (0.89–0.95)	0.80 (0.75–0.86)	0.600	0.000
Yes	16	1.36 (0.42–4.39)	0.602	0.736	0.86 (0.69–1.00)	0.75 (0.53–1.00)		
**Uterine fibroids**^**a**^								
No	318	–			0.92 (0.89–0.95)	0.80 (0.75–0.86)	0.039	0.000
Yes	17	2.57 (1.02–6.49)	0.046	0.170	0.81 (0.64–1.00)	0.71 (0.50–1.00)		
**Polycystic ovary disease**^**a**^								
No	274	–			0.92 (0.88–0.95)	0.81 (0.76–0.87)	0.450	0.000
Yes	61	1.31 (0.65–2.64)	0.447	0.639	0.91 (0.83–0.99)	0.75 (0.62–0.91)		
**Adenomyosis**^**a**^								
No	315	–			0.93 (0.90–0.96)	0.81 (0.76–0.87)	0.002	0.350
Yes	20	3.28 (1.47–7.32)	0.004	0.031	0.67 (0.48–0.92)	0.60 (0.41–0.88)		
**US—uterine volume (ml)**								
Overall	141	1.01 (1.00–1.02)	0.001	0.008				~
Under 40 ml	17				0.88 (0.73–1.00)	0.79 (0.60–1.00)	0.027	
40–110 ml	107	–			0.90 (0.84–0.96)	0.68 (0.58–0.80)		
> 110 ml	17				0.61 (0.41–0.92)	0.34 (0.13–0.89)		
**US—log uterine volume (ml)**								
Overall	141	7.72 (1.53–39.07)	0.013	0.067	–	–		~
**US—fibroids present**								
No	130	–			0.87 (0.81–0.93)	0.70 (0.61–0.81)	0.230	~
Yes	21	1.62 (0.74–3.55)	0.231	0.413	0.85 (0.71–1.00)	0.57 (0.37–0.90)		
**US—ovarian cysts present**								
No	98				0.90 (0.84–0.96)	0.70 (0.59–0.83)	0.430	~
Yes	53	1.30 (0.67–2.53)	0.434	0.639	0.82 (0.71–0.93)	0.63 (0.49–0.82)		
**US—polycystic ovaries present**								
No	134	–			0.85 (0.79–0.92)	0.66 (0.57–0.77)	0.150	~
Yes	17	0.37 (0.09–1.54)	0.171	0.347	1.00 (1.00–1.00)	0.85 (0.67–1.00)		
**US—adenomyosis present**								
No	115	–			0.88 (0.83–0.95)	0.71 (0.62–0.82)	0.068	~
Yes	36	1.90 (0.94–3.81)	0.072	0.213	0.82 (0.70–0.96)	0.55 (0.38–0.82)		
**US—adenomyosis linear striations present**								
No	135	–			0.86 (0.80–0.93)	0.69 (0.60–0.80)	0.700	~
Yes	16	1.20 (0.47–3.09)	0.705	0.822	0.93 (0.82–1.00)	0.61 (0.40–0.95)		
**US—adenomyosis heterogeneous myometrium**								
No	141	–			0.88 (0.82–0.93)	0.71 (0.62–0.81)	0.003	~
Yes	10	3.92 (1.48–10.38)	0.006	0.038	0.80 (0.59–1.00)	0.00 (NA–NA)		
**US—adenomyosis thickened posterior wall**								
No	135	–			0.89 (0.83–0.94)	0.69 (0.60–0.79)	0.093	~
Yes	16	2.23 (0.85–5.83)	0.102	0.245	0.72 (0.52–1.00)	0.72 (0.52–1.00)		
**Pre-cancer of the cervix or an abnormal Papanicolaou test**^**a**^								
No	319	–			0.92 (0.88–0.95)	0.80 (0.75–0.86)	0.960	0.000
Yes	16	1.03 (0.32–3.33)	0.955	0.960	0.93 (0.82–1.00)	0.79 (0.61–1.00)		
**Cervical intraepithelial neoplasia (CIN) (pathology)**								
No	298	–			0.92 (0.89–0.95)	0.81 (0.76–0.87)	0.260	0.000
Yes	37	1.55 (0.72–3.31)	0.260	0.444	0.89 (0.79–1.00)	0.69 (0.52–0.91)		
**Food allergies or intolerances**^**a**^								
No	258	–			0.93 (0.89–0.96)	0.79 (0.73–0.86)	0.840	0.000
Yes	77	0.93 (0.48–1.83)	0.845	0.901	0.88 (0.81–0.96)	0.81 (0.72–0.93)		
**Disturbance to taste or smell**^**a**^								
No	312	–			0.92 (0.89–0.96)	0.82 (0.77–0.87)	0.023	0.000
Yes	23	2.36 (1.10–5.04)	0.027	0.110	0.82 (0.67–1.00)	0.62 (0.44–0.87)		
**Hay fever**^**a**^								
No	206	–			0.93 (0.90–0.97)	0.81 (0.74–0.89)	0.310	0.000
Yes	129	1.34 (0.76–2.36)	0.310	0.507	0.89 (0.83–0.95)	0.78 (0.70–0.87)		
**Eczema**^**a**^								
No	236	–			0.91 (0.87–0.95)	0.77 (0.70–0.84)	0.089	0.000
Yes	99	0.55 (0.27–1.11)	0.094	0.242	0.93 (0.88–0.99)	0.87 (0.80–0.95)		
**Previous diagnosis of endometriosis**^**a**^								
No	306	–			0.97 (0.96–0.99)	0.90 (0.86–0.95)	< 0.001	~
Yes	29	32.46 (17.69–59.59)	0.000	< 0.001	0.38 (0.24–0.60)	0.03 (0.01–0.24)		
**Family history of endometriosis**^**a**^								
No	252	–			0.91 (0.87–0.95)	0.78 (0.72–0.85)	0.380	0.000
Yes	82	0.73 (0.37–1.48)	0.386	0.593	0.94 (0.88–0.99)	0.84 (0.75–0.94)		
**US—endometriosis present (excluding OMA)**								
No	137	–			0.90 (0.85–0.96)	0.73 (0.64–0.83)	< 0.001	~
Yes	14	6.26 (2.99–13.09)	0.000	< 0.001	0.55 (0.34–0.90)	0.24 (0.08–0.73)		
**US—endometrioma**								
No	107	–			0.91 (0.85–0.97)	0.71 (0.61–0.83)	0.150	~
Yes	44	1.63 (0.83–3.18)	0.154	0.330	0.78 (0.66–0.92)	0.59 (0.44–0.80)		
**US—adhesions**								
No	136	–			0.89 (0.84–0.95)	0.71 (0.62–0.82)	0.001	~
Yes	15	3.32 (1.56–7.05)	0.002	0.018	0.67 (0.47–0.95)	0.44 (0.25–0.80)		
**rASRM score**								
Overall	335	1.02 (1.01–1.02)	0.000	< 0.001				0.000
**Stage of endometriosis (rASRM)**								
Stage 1	192	–			0.97 (0.94–1.00)	0.91 (0.86–0.96)	< 0.001	
Stage 2	53	3.40 (1.47–7.88)	0.004	0.033	0.89 (0.80–0.99)	0.71 (0.56–0.89)		0.000
Stage 3	37	3.44 (1.35–8.76)	0.010	0.057	0.91 (0.82–1.00)	0.64 (0.44–0.94)		0.000
Stage 4	53	6.79 (3.29–14.00)	0.000	< 0.001	0.76 (0.65–0.89)	0.59 (0.46–0.77)		0.000
**Adhesions (surgical)**								
No	201	–			0.97 (0.95–1.00)	0.92 (0.88–0.97)	< 0.001	0.570
Yes	134	5.49 (2.80–10.76)	0.000	< 0.001	0.84 (0.77–0.90)	0.63 (0.54–0.74)		
**Superficial ovarian lesion(s)**								
No	289	–			0.92 (0.89–0.96)	0.82 (0.76–0.87)	0.140	0.000
Yes	46	1.72 (0.83–3.56)	0.143	0.315	0.88 (0.78–0.98)	0.69 (0.52–0.91)		
**Deep ovarian lesion(s)**								
No	253	–			0.93 (0.90–0.96)	0.84 (0.78–0.90)	0.013	0.000
Yes	82	2.06 (1.15–3.67)	0.015	0.067	0.88 (0.81–0.96)	0.68 (0.56–0.83)		
**Superficial peritoneal lesion(s)**								
No	45	–			0.84 (0.74–0.96)	0.67 (0.54–0.84)	0.009	0.000
Yes	290	0.44 (0.23–0.83)	0.011	0.060	0.93 (0.90–0.96)	0.82 (0.77–0.88)		
**Deep peritoneal lesion(s)**								
No	230	–			0.97 (0.95–1.00)	0.84 (0.78–0.91)	< 0.001	0.000
Yes	105	3.03 (1.72–5.35)	0.000	0.002	0.79 (0.72–0.88)	0.71 (0.62–0.82)		
**Pouch of Douglas**								
No	142	–			0.94 (0.89–0.98)	0.82 (0.74–0.91)	0.390	0.000
Yes	193	1.30 (0.71–2.38)	0.389	0.593	0.90 (0.86–0.95)	0.79 (0.72–0.86)		
**UV pouch**								
No	246	–			0.92 (0.89–0.96)	0.82 (0.76–0.88)	0.210	0.000
Yes	89	1.46 (0.80–2.67)	0.214	0.393	0.89 (0.83–0.97)	0.75 (0.64–0.87)		
**Bladder**								
No	333	–			0.92 (0.89–0.95)	0.80 (0.75–0.86)	0.042	0.000
Yes	2	6.15 (0.83–45.33)	0.075	0.213	0.50 (0.13–1.00)	0.50 (0.13–1.00)		
**Bowel**								
No	280	–			0.96 (0.94–0.99)	0.86 (0.81–0.92)	< 0.001	1.039
Yes	55	6.25 (3.54–11.03)	0.000	< 0.001	0.67 (0.55–0.82)	0.48 (0.33–0.67)		
**Tubes**								
No	324	–			0.92 (0.89–0.95)	0.80 (0.74–0.85)	0.700	0.000
Yes	11	0.76 (0.18–3.12)	0.699	0.822	0.87 (0.72–1.00)	0.87 (0.72–1.00)		
**Pelvic side wall**								
No	157	–			0.92 (0.87–0.98)	0.85 (0.77–0.93)	0.550	0.000
Yes	178	1.22 (0.64–2.30)	0.546	0.689	0.91 (0.88–0.95)	0.78 (0.72–0.85)		
**Pararectal space**								
No	307	–			0.91 (0.87–0.94)	0.80 (0.74–0.86)	0.450	0.000
Yes	28	0.67 (0.24–1.87)	0.448	0.639	0.97 (0.92–1.00)	0.80 (0.64–1.00)		
**Uterosacral ligament**								
No	211	–			0.93 (0.89–0.97)	0.79 (0.72–0.87)	0.750	0.000
Yes	124	0.91 (0.51–1.62)	0.747	0.836	0.90 (0.86–0.96)	0.82 (0.74–0.90)		
**Other**								
No	245	–			0.92 (0.89–0.96)	0.82 (0.76–0.88)	0.086	0.000
Yes	90	1.67 (0.92–3.03)	0.089	0.237	0.89 (0.83–0.96)	0.75 (0.64–0.88)		

~ Data was not included in the multivariate Lasso model

^a^Patient reported variable

Several features were found to be significant in the survival analysis, but only 3 features demonstrated robust non-zero Lasso coefficients: bowel lesions (1.039), adhesions (0.570) and adenomyosis (0.350) (Table 4). The presence of lesions on the bowel was highly discriminatory and increased the risk for recurrent disease (univariate HR 6.25 [3.54–11.03], adj. *p* value < 0.001) (Table 4 and Fig. 4a). At 2 years following their first diagnostic surgery, patients with bowel lesions have a 67% probability of no recurrence compared to a much higher 96% probability of no recurrence in those without bowel lesions (Table 4 and Fig. 4a). At 5 years, the probability of no recurrence drops to 48% in patients with bowel lesions versus 86% in those without bowel lesions. The presence of adhesions visualised at the time of diagnosis was similarly highly predictive of recurrent disease. Adhesions increased the odds of recurrence (univariate HR 5.49 [2.80–10.76], adj. *p* value < 0.001) (Table 4 and Fig. 4c). Two years following diagnostic surgery, patients with adhesions have an 84% probability of no recurrence compared to 97% probability of no recurrence in those without adhesions (Table 4 and Fig. 4c). At 5 years, the probability of no recurrence drops to 63% in patients with adhesions versus a still relatively high 92% in those without adhesions. Reporting adenomyosis at the time of surgery was also predictive of disease recurrence (univariate HR 3.28 [1.47–7.32], adj. *p* value 0.031), and by 5 years, the probability of no recurrence in those with adenomyosis was 60% compared to 81% in those who did not report adenomyosis (Table 4 and Fig. 4b).

## Discussion

The aetiology of recurrent endometriosis is poorly understood, which is problematic since disease recurrence impacts more than 60% of patients (reviewed by 1, 2). Here, we identify risk factors for endometriosis recurrence using clinical and survey information from cases and non-endometriosis controls. Multiple factors were identified that increase the risk of developing recurrent disease; however, 3 key features—adenomyosis, bowel lesions and adhesions—were repeatedly associated with endometriosis recurrence. The findings of this study may be helpful for the clinical management of endometriosis and reducing rates and burden of recurrence.

Presence of self-reported adenomyosis was repeatedly associated with recurrent endometriosis. Adenomyosis also contributed to an increased risk score for predicting recurrence and significantly increased probability of recurrence 2 and 5 years following an endometriosis diagnosis. Non-invasive diagnostic tests for adenomyosis are improving, with imaging procedures including transvaginal ultrasound and magnetic resonance imaging (MRI) accurately reflecting histological findings [21, 22]. Using our clinically derived ultrasound data, we observed an increased risk of recurrence relative to non-endometriosis controls for presence of adenomyosis, as well as increased uterine volume. We have previously reported that ultrasound diagnosed adenomyosis is associated with stage 4 endometriosis [23]. As the symptoms of adenomyosis commonly mimic endometriosis, it is very probable that individuals are undergoing reoperation for treatment of suspected recurrent endometriosis, when in fact the primary cause of recurring symptoms is adenomyosis. This is a scenario that may have occurred in our study. Others have reported a relationship between adenomyosis of the outer uterine wall and bowel endometriosis [24, 25]. Recently, it has been highlighted that nodules in the bowel may not be caused by deep-infiltrating endometriosis, but rather the result of posterior uterine adenomyosis [26]. It is suggested that because of the close proximity between posterior wall adenomyosis and the bowel, it could attach to and seed in the adjacent colon, or vice versa, and increase the likelihood of adhesions obliterating the pouch of Douglas [25].

We found that endometriotic bowel lesions were associated with higher risk of disease recurrence, especially in surgically confirmed recurrent patients. Bowel lesions are present in 3–37% of all endometriosis patients [27] and are more common on the sigmoid colon and rectum [25]. Endometriosis of the bowel may be solitary, but often presents with ovarian or uterosacral ligament endometriosis nodules and adenomyosis [24]. In the context of the overall findings of our study, it is acknowledged that bowel endometriosis is reported to be associated with advanced pelvic adhesions and fibrosis [25, 28]. Therefore, a picture is emerging where an unfavourable paradigm of adenomyosis, bowel lesions and adhesions co-exist to increase the severity of disease and promote an environment favourable for endometriosis recurrence. There are reports in the literature of patients with bowel endometriosis developing recurrence; however, these are often case reports [29–31] and cannot be accurately compared to recurrence rates of other subtypes of endometriosis (e.g. peritoneal lesions or endometrioma). One systematic review reported surgically confirmed endometriosis recurrence to be 13.9% following bowel resection [29]. A second systematic review showed higher recurrence rates (visually and/or histologically proven recurrence) of 45% following bowel resection anastomosis and 35% following other surgical methods (shaving, superficial excision, full thickness disc excision) [32]. We did not collect information pertaining to the type of procedure performed for removal of bowel disease, therefore cannot speculate if our cohort was different or similar to the report of Meuleman et al. Medical management of bowel endometriosis can lead to symptom recurrence following discontinuation of therapy, and therefore, surgical management is considered to be the primary treatment for symptomatic severe endometriosis of the bowel [32]. Yet, a histological study of 10 patients who underwent colorectal resection, with edges ≥ 2 cm away from the macroscopic nodule limits, identified microscopic endometriosis in the colorectal muscularis layer adjacent and distal from the macroscopic nodule limits [24]. Therefore, it is important to raise the question of feasibility of complete resection of bowel endometriosis if microscopic implants remain in patients managed by segmental resection [24].

Notwithstanding, the possibility of microscopic endometriosis remaining following resection or bowel shaving, lesions on the bowel are often not removed or incompletely removed due to clinical considerations. The reasons for this vary and may be anecdotal, but operative morbidity may take priority over complete disease resection [27]. The current definition of recurrent endometriosis specifies ‘lesion recurrence (…) after previous complete excision of the disease’ [7]; this presents a limitation to many published works as description of complete/incomplete excision is often not provided. Therefore, it is difficult to ascertain if studies describing recurrent endometriosis are actually describing persistent or residual endometriosis instead of de novo disease. One study quantified the risk of recurrence after complete resection of endometriosis, versus leaving residual disease and found while total resection did decrease the risk of disease recurrence, it increased the risk of post-surgical complications and was reliant on surgeon expertise [33]. In our study, we took steps to ensure those with incomplete resection or abandoned surgeries were not included in the subset of patients classified as having recurrent disease, and therefore, we can infer that presence of bowel disease at the index surgery is associated with de novo lesion recurrence, not due to disease that was left behind in previous surgeries.

One consideration that will influence a surgeon’s decision regarding complete removal of endometriosis and increased likelihood of reoperation is post-surgical adhesions. The risk of adhesions after any abdominopelvic operation has previously been reported to be between 55 and 100% [34]. Presence of adhesions was significantly associated with endometriosis disease recurrence in the current study. The observed adhesions in our participants were likely a result of the inflammatory endometriotic lesions themselves or from their previous surgery or surgeries.

Strategies to reduce adhesion formation following surgery have been the topic of two recent Cochrane reviews examining the effectiveness of barrier or gel agents, which report scanty evidence demonstrating effectiveness in adhesion prevention [35] or low-quality evidence supporting barrier agents in reducing adhesion formation [36]. New data is emerging on novel treatments for adhesions [37, 38], as well as non-invasive methods of detecting adhesions, such as computed tomography (CT) imaging [39]. One study showed treatment with intraperitoneal triamcinolone (a glucocorticoid used to treat skin conditions and autoimmune disorders) following gynaecological surgery reduced adhesions at a repeat abdominal surgery [38]. With respect to adhesion detection via imaging for diagnosis or as part of pre-surgical planning, our data demonstrated that ultrasound was useful in identifying adhesions in association with disease recurrence. Thus, increased surveillance and imaging should be considered the first-line approach to assess the presence of adhesions when suspicious of endometriosis recurrence, as opposed to performing multiple surgeries, which may increase the risk of further adhesions and recurrent disease.

Common biological mechanisms tie adhesions, adenomyosis and bowel lesions together—inflammation, fibrosis and epithelial-to-mesenchymal transition (EMT). These pathways are ingrained in many of the pathophysiological theories of endometriosis lesion development and survival and have been extensively reviewed [40–43]. A deeper understanding of these mechanisms, specific to their contribution to disease recurrence, may provide researchers and clinicals with an opportunity to develop targeted therapies that could be utilised to prevent disease from returning.

In this study, a positive diagnosis of endometriosis (recurrent disease or first diagnosis) was associated with decreased likelihood of hormone medication usage compared to non-endometriosis controls. A recent publication reported reduced reoperation for endometriosis in patients who are treated with hormones before and after their first endometriosis surgery, with the median time to reoperation estimated to be 9 years compared to just 3 years in those not taking hormone therapy [44]. However, a 2020 Cochrane Review (of 25 trials) exploring the effectiveness of hormonal suppression before, after or both before and after surgery for endometriosis determined that the data was inconclusive, stating that those ‘who receive postsurgical medical therapy compared with no medical therapy or placebo *may* experience benefit in terms of (…) disease recurrence (…). There is insufficient evidence regarding hormonal suppression therapy at other time points in relation to surgery for women with endometriosis’ [45]. It is not known why our population were less likely to report use of hormones, and while it could be speculated that a proportion were trying to conceive, we cannot report that with certainty. It is clear, however, that more comprehensive prospective studies are required to unequivocally answer if hormonal suppression can prevent endometriosis recurrence.

In addition to the characteristics described above, some other factors were found to be associated with an increased risk of recurrence (and increased risk score) and arose on more than one occasion. The relationship between endometriosis and reduced gravidity and parity is well-established [46, 47]. However, in our study, recurrent endometriosis patients had increased odds of higher gravidity and/or parity compared to those with a single diagnosis of endometriosis. It is likely this phenomenon is age-related, with recurrent patients more likely to be older than those receiving a single diagnosis and therefore, having greater amount of time to achieve more pregnancies. This is supported by our recent study that found the likelihood of a new diagnosis of endometriosis in those with pelvic pain, no previous laparoscopy and a normal ultrasound, was lower in women aged 40 and above [48]. On the other hand, patients of greater age and higher gravidity and parity may be more likely to accept reoperation as a treatment and elect for a hysterectomy, as the desire for future fertility has lapsed, thus falling into the recurrence cohort in this analysis.

A lower BMI has commonly been associated with increased risk of endometriosis [49–51]. However, following stratification of our endometriosis groups into recurrent and single diagnoses, we found that obesity was associated with an increased likelihood of endometriosis recurrence compared to patients with a single diagnosis of endometriosis (in the surgically confirmed analysis only). Interestingly, we also found an association between recurrent endometriosis and increased blood pressure compared to those with a single diagnosis. Yet, when recurrent endometriosis was compared to non-endometriosis controls, the dynamic shifted and obesity became protective. In other words, obesity reduced the risk of recurrent endometriosis compared to controls. The presence of worsening metabolic disease in endometriosis is well established; evidence in the literature describes high blood pressure, hypercholesterolemia, fasting glucose levels and cardiovascular disease in endometriosis patients over time [52–54]. Critically, there is an established relationship between chronic pain and the development of hypertension [55, 56], and the risk of cardiovascular disease [57]. Though this requires further investigation, we suggest that recurrent endometriosis may represent a cohort of patients with an increased propensity for the development of cardiometabolic diseases (including obesity and hypertension), and that these patients warrant long-term surveillance and monitoring in order to reduce cardiometabolic risk.

Inherently, it is perceived that data from self-reported surveys are limited, as information can suffer from response bias or recall bias. However, recent studies have found that patients who self-report a previous endometriosis diagnosis do so with accuracy [58, 59]. In our study, almost 80% of patients with surgically confirmed recurrent endometriosis also self-reported a previous diagnosis, validating that a patient’s account of a positive diagnosis of endometriosis should be considered reliable, in the absence of any confirmatory medical documentation.

This study considered the presence of de novo lesions following index surgery a pre-requisite for categorisation into the recurrent endometriosis group. Yet, to achieve this status, it must be kept in mind that multiple likely events were necessary in the lead up to reoperation. For example, the individual must have experienced symptom recurrence following a previous surgery or have been unresponsive to non-surgical management (i.e. hormonal treatment) of returning symptoms. However, not all individuals will elect non-surgical management, for example, if they have had a prior negative experience with the side effects of hormonal therapies and a positive experience of symptom relief following surgery. In addition, repeat surgery must be consistent with the patient’s desire as well as the surgeon’s/hospital’s assessment and practice. All of these aforementioned decisions are complex and subjective, and further randomised controlled clinical trials (RCTs) are necessary to overcome and better understand these potential biases.

The strengths of this study include its novelty, size, comprehensive clinical phenotyping and multivariate analysis. Furthermore, our inclusion of two analytic groups defining endometriosis recurrence increases the robustness of the findings. However, we acknowledge the following limitation, while the self-reported analysis utilised pathology-confirmed endometriosis in the selection and allocation of participants, the surgically confirmed analysis relied on screening of surgical records, therefore surgical visualisation. While the outcomes of this research bring us closer to developing a clinical model to predict the chance of recurrence for those diagnosed with endometriosis, the accuracy of these tools are not yet precise enough to correctly classify disease outcomes. The prediction model is highly influenced by the samples on which it was developed (in this case, patients with significant pelvic pain or symptoms of endometriosis that warranted surgery in a tertiary hospital), and therefore, generalising to other populations may be less accurate. Further external validation in more diverse populations is necessary. We also expected to observe more replication of features between our two study analyses, self-reported and surgically confirmed endometriosis recurrence. We may have seen improved replication or more accurate risk prediction if we had further stratified repeat surgeries by procedure type, particularly, the involvement of a hysterectomy (with/without ovarian conservation). Hysterectomy at reoperation is a notable consideration, given this larger procedure is associated with reduced risk of recurrence [2, 60, 61]. We have specifically examined endometriosis cases (single and recurrent) attributed by the physical presence of lesions, visualised at surgery, and have not considered symptom-based suspected recurrence. The mechanisms of symptom only recurrence versus new lesion formation are likely to have very different aetiologies, and therefore, interpretation of the findings of this investigation should only be considered in the context of lesions being present. However, as our focus was on surgical patients, our study did not include patients with a clinical diagnosis of recurrence (for example, recurrence found on imaging [ultrasound or MRI] or diagnostic laparoscopy alone) who were managed non-surgically. A final limitation of our study is time; it would have been beneficial to follow participants for a longer follow-up period to monitor for disease recurrence and the long-term effectiveness of interventions.

## Conclusions

A picture has emerged that highlights several features to be important for better understanding the mechanisms behind recurrent endometriosis. A negative paradigm consisting of lesions located on the bowel, post-surgical adhesions and adenomyosis are observed to increase the severity of disease and the risk of endometriosis recurrence. This information has been derived from a combination of patient reported measures and clinical records, highlighting and validating the use of self-reported patient histories for prior diagnoses of endometriosis. In the course of diagnosing suspected recurrent disease, the presence of other endometriosis features (lesions on the bowel, adhesions) and gynaecological comorbidities (adenomyosis) should also be accurately assessed at the same time [44]. Being able to provide patients more comprehensive information on the likelihood of their disease returning following complete surgical removal will empower patients with knowledge and a level of reassurance not previously available. While further work is needed to validate our tool and its predictive power, the current study provides evidence of clinically detectable risk factors associated with an increased chance of disease recurrence.

## Data Availability

Individual participant data (including data dictionaries) are not available for sharing.  The ethics approvals of this study prohibit the study team from making the dataset publicly available.

## Abbreviations

AUC: Area under the curve
BMI: Body mass index
BP: Blood pressure
CI: Confidence interval
CIN: Cervical intraepithelial neoplasia
cm: Centimetre
CT: Computed tomography
CV: Cross-validation
EMT: Epithelial-to-mesenchymal transition
ER: Emergency room
HR: Hazards ratio
MRI: Magnetic resonance imaging
*n*: Number
NHMRC: National Health and Medical Research Council
OR: Odds ratio
rASRM: Revised American Society for Reproductive Medicine
RCT: Randomised controlled clinical trial
RiskSLIM: Risk-calibrated Supersparse Linear Integer Models
ROC: Receiver operating characteristic
RWH: Royal Women’s Hospital
STROBE: STrengthening the Reporting of OBservational studies in Epidemiology
UV: Uterovesical
